# Independent and interactive effects of wet bulb globe temperature and air pollution exposures on suicide mortality

**DOI:** 10.1016/j.envint.2026.110152

**Published:** 2026-02-21

**Authors:** Dirga Kumar Lamichhane, James A. VanDerslice, Fred Lurmann, Nathan R. Pavlovic, Michael J. Staley, Douglas S. Tharp, Alina Peluso, Brandy M. Byrwa-Hill, Yue Zhang, Anna R. Docherty, Hilary Coon, Amanda V. Bakian

**Affiliations:** a Department of Psychiatry, Spencer Fox Eccles School of Medicine, University of Utah, Salt Lake City, UT, USA; b Huntsman Mental Health Institute, University of Utah, Salt Lake City, UT, USA; c Division of Public Health, Department of Family Medicine and Public Health, Spencer Fox Eccles School of Medicine, University of Utah, Salt Lake City, UT, USA; d Sonoma Technology, Inc., Petaluma, CA, USA; e Utah State Office of the Medical Examiner, Utah Department of Health and Human Services, Salt Lake City, UT, USA; f Oak Ridge National Laboratory, Oak Ridge, TN, USA; g Division of Epidemiology, Department of Internal Medicine, Spencer Fox Eccles School of Medicine, University of Utah, Salt Lake City, UT, USA; h Division of Biostatistics, Department of Population Health Science, Spencer Fox Eccles School of Medicine, University of Utah, Salt Lake City, UT, USA

**Keywords:** Wet bulb globe temperature, Nitrogen dioxide, Fine particulate matter, Suicide mortality, Season, Interaction

## Abstract

**Background:**

Individual components of the ambient environment, such as temperature and air pollution, exist as part of a complex mixture and have been associated with suicide; however, their interactive effects remain poorly understood. This study examined the independent and interactive effects of wet bulb globe temperature (WBGT), nitrogen dioxide (NO_2_), and fine particulate matter (PM_2.5_) on suicide mortality.

**Methods:**

We identified 7,551 suicide cases in Utah, USA, from 2000 to 2016 and assigned exposure to daily maximum WBGT (sourced from the European Center for Medium-Range Weather Forecasts) and PM_2.5_ and NO_2_ concentrations (sourced from a national spatiotemporal ensemble model) using decedent’s residential address at the time of death. A case-crossover design with conditional logistic regression was used to estimate the independent and interactive effects of WBGT_max_, PM_2.5_, and NO_2_ on suicide. For exposure windows, we considered single days preceding suicide (lag 0 to 6) and their averages across preceding days (lag 0–1, 0–3, and 0–6) Analyses were stratified by season.

**Results:**

We identified a significant association between WBGT_max_ and suicide across all seasons (odds ratio [OR] = 1.05, 95% confidence interval [CI]: 1.01, 1.10; per 5 °C increase on lag 0–3 days). The associations were stronger in the warm season (March 22 to September 21), with ORs and 95% CIs ranging from 1.08 (1.02, 1.15) to 1.20 (1.10, 1.30) per 5 °C increase depending on the lag periods. We observed synergistic interactions between WBGT_max_ and PM_2.5_ and NO_2_ in the warm season, associated with higher odds of suicide. The associations of WBGT_max_ with suicide were most pronounced at high NO_2_ levels.

**Conclusions:**

We found evidence of synergistic interactions between WBGT_max_ and PM_2.5_ and NO_2_ on suicide in the warm season, emphasizing the need for considering the combined effects of heat stress and air pollution in suicide prevention strategies.

## 1. Introduction

In 2022, over 49,000 individuals died by suicide in the U.S., making it the 11th leading cause of death (Garnett and Curtin, 2024). The age-adjusted suicide rate in Utah in 2022 (22.1 per 100,000 people) was higher than the national average (14.2 per 100,000 people) and was the state’s 9th leading cause of death (Centers for Disease Control and Prevention, 2024; Garnett and Curtin, 2024). In addition to genetic factors, a combination of proximal and distal environmental factors is known to contribute to suicide (Coon et al., 2020; Turecki et al., 2019). The proximal period prior to suicide is a critical window for intervention, yet the ability to predict who is most at risk of suicide remains poor (Belsher et al., 2019; Esposti and Kaufman, 2023). Growing evidence indicates that factors in the ambient environment such as air pollution and weather conditions may serve as short-term risk factors for suicide (Bakian et al., 2015; Molitor et al., 2023; Rahman et al., 2023). A thorough understanding of these factors and their interactions is essential for developing effective suicide prevention strategies.

Proximal exposure to weather factors, such as temperature and solar radiation, and air pollutants, including particulate matter with an aerodynamic diameter of ≤ 2.5 μm (PM_2.5_) and nitrogen dioxide (NO_2_), have been associated with suicide risk in studies conducted in different geographical locations including the U.S., Europe, and East Asia (Frangione et al., 2022; Heo et al., 2021). However, these studies have focused on examining the effect of a single exposure while adjusting for other environmental exposures and potential confounders. In normal ambient conditions, people are exposed to multiple air pollutants and meteorological factors simultaneously, with their health impacts potentially manifesting through moderating or interactive effects due to their interdependent and linked relationships (Yitshak-Sade et al, 2018). For example, while meteorological factors and air pollutants co-occur in a mixture, weather conditions also impact air pollutant concentrations through chemical reactions, transport, mixing of atmospheric components, and deposition processes (Im et al., 2022; Shen et al., 2021). In addition, weather conditions vary seasonally, which impacts air pollutants’ mixing heights and, correspondingly, their concentrations, with the highest mixing heights observed in the summer and the lowest observed in the winter (Ahmad et al., 2024). Differential air pollutant concentrations across the seasons compounded with temperature exposures could differentially affect health outcomes.

Although several weather factors have been associated with increased all-cause mortality risk, including precipitation, humidity, and solar radiation, the effects of ambient temperature have received the most consideration. High temperature and air pollutants have been found to interact in the short term to increase the risk of all-cause, respiratory, and cardiovascular mortality (Areal et al., 2022; Lee et al., 2019; Rahman et al., 2022; Stafoggia et al., 2023). Regarding suicide, only a couple of prior studies have examined if air pollution interacts with temperature to influence the risk of suicide mortality-related outcomes (Kim et al., 2024; Villeneuve et al., 2023). Prior work has generally used a single meteorological variable, such as mean or maximum daily air temperature, as the primary indicator of heat stress (Lee et al., 2019; Stafoggia et al., 2023). However, ambient temperature alone may not effectively quantify the heat stress experienced by the human body, as it neglects key contributors to thermal load, including humidity and solar radiation. Wet bulb globe temperature (WBGT) is an apparent heat measure that integrates air temperature, humidity, wind speed, and solar radiation, and may better reflect the heat stress experienced by individuals in a thermal environment where multiple weather factors interact (Andrews et al., 2018; Liljegren et al., 2008). Although previous studies have examined the independent effects of short-term temperature and humidity exposures on suicide (Florido et al., 2021; Rahman et al., 2023), no research has considered a composite index of heat stress including WBGT.

In addition to the lack of knowledge on synergistic environmental effects, much of the existing work on environmental contributors to suicide risk has been done outside the U.S., with the work focusing on temperature but not apparent heat. To fill this knowledge gap, we conducted a bi-directional, time-stratified case-crossover study of suicide mortality in Utah, USA, from 2000 to 2016. As with many parts of the world, climate change-related increases in air pollution and temperature are significant in the U.S. (Chikamoto et al., 2023; McClure and Jaffe, 2018). Particularly, in Utah, the mountain-valley topography, combined with meteorological conditions, leads to significant temporal variability in air pollution (Ou et al., 2020). Utah has distinct weather conditions by season, and Utah’s high mountain valleys may experience serious air pollution events in winter, mostly driven by multi-day temperature inversions (Lyman and Tran, 2015; Silva et al., 2007). These unique environmental characteristics make Utah an important setting for the exploration of the associations between heat stress and air pollution with suicide. In this study, we aim to investigate the independent and interactive associations of WBGT and air pollutants (PM_2.5_ and NO_2_) with suicide mortality. In addition, as prior Utah work found stronger associations of PM_2.5_ and NO_2_ with suicide in the spring/fall transition period (Bakian et al., 2015) and because of the seasonal variability in the distribution of air pollutants and suicide risk (Peng et al., 2005; To et al., 2024), we aimed to determine how these associations varied by season.

## 2. Methods

### 2.1. Study population

The study population included all suicide deaths in Utah from January 1, 2000 through December 31, 2016. Suicide deaths were ascertained through a long-standing collaboration between the Utah Suicide Mortality Research Study (USMRS) and the Utah Department of Health and Human Service’s Office of the Medical Examiner (OME). Upon suspecting a suicide death, the OME performed a thorough investigation and sent demographic data (e.g., date of death, age, and sex) to an honest broker unconnected with the analysis team within the Utah Population Database (UPDB) (Coon et al., 2020; Docherty et al., 2020). The UPDB is an unprecedented data resource that houses or links to several administrative datasets for Utah residents including vital records, genealogical records, and health billing codes (Smith and Fraser, 2018). After receiving the data from OME, the UPDB securely linked suspected suicides to their death certificates to confirm suicide as the cause of death. The residential address at the time of death was acquired from death certificates and was geocoded to latitude and longitude by the UPDB. The study was approved by the Utah Resource for Genetic and Epidemiologic Research and received exempt status from the Institutional Review Board of the University of Utah (#00144844).

### 2.2. Environmental data

We obtained high-resolution daily PM_2.5_ (24-hour average) and NO_2_ (1-hour maximum) concentrations at the suicide decedent’s residential death certificate address from spatiotemporal ensemble models that estimated ambient concentrations at 1 km^2^ grid resolution for the entire U.S. from 2000 to 2016 (Di et al., 2019 and 2020). Individual air pollution exposure assignments were based on the gridded values.

High spatial resolution (approximately 9 km) hourly ambient WBGT estimates across Utah from 2000 to 2016 were calculated at the decedent’s residential locations using the European Center for Medium-Range Weather Forecasts (ECMWF) Reanalysis v5 – Land dataset (ERA5-Land) (C3S, 2019; Muñoz-Sabater et al., 2021). WBGT is a physiologically motivated heat-stress index that explicitly integrates air temperature, wind speed, humidity, and radiant heat. We chose to focus on 1-hour maximum WBGT (WBGT_max_) versus other heat or heat stress measures because it is consistent with occupational heat standards, captures peak heat stress, and is sensitive to radiation and wind, which are key drivers of heat strain (Spangler et al., 2022). We calculated WBGT using the Liljegren approach as implemented by Spangler et al. (2022). Specific details on the WBGT calculation can be found in Text S1. In the present study, we used daily 1-hour WBGT_max_ rather than daily minimum WBGT or mean WBGT, as the former has been found to exert a greater influence on human health (Spangler et al., 2023).

### 2.3. Study design

We used a bi-directional time-stratified case-crossover study design to investigate the independent and interactive effects of short-term exposure to air pollutants and WBGT_max_ on the risk of suicide in Utah from 2000 to 2016. This study design has been widely used to investigate the health risks associated with short-term exposure to air pollution and temperature and has previously been used to analyze the associations of short-term environmental exposures with suicide mortality (Bakian et al., 2015; Villeneuve et al., 2023). We defined a case day as the day of the suicide death and selected 3 or 4 control days that were matched based on the same day of the week, month, and year as the case day (e.g., if the suicide death occurred on a Sunday in April 2016, then all other Sundays in April 2016 were selected as the control days). This method controls for potential confounding effects by the day of the week, seasonality, and long-term trends, as well as for all potential individual-level confounders that were unlikely to vary in the short term.

### 2.4. Statistical analysis

We calculated summary statistics for demographic characteristics and environmental exposures (WBGT_max_, PM_2.5_, and NO_2_) by WBGT_max_ levels and season. The seasons were defined as warm (March 22 to September 21) and cold (September 22 to March 21) using astronomical cut points. We used t-tests to test for differences in the distributions of environmental exposures between the case and control periods, and Pearson’s correlations were estimated to measure the associations among exposures. Violin plots were used to visualize exposure and death count distributions.

Conditional logistic regression (CLR) models were formulated to estimate the odds ratios (OR) and corresponding 95% confidence intervals (CI) of suicide death associated with a 10-unit increase in PM_2.5_ (μg/m^3^) or NO_2_ (ppb) and a 5-unit increase in WBGT_max_ (°C). We used these increments to facilitate comparisons across studies and to represent potentially policy-relevant shifts. We investigated WBGT_max_, PM_2.5_, and NO_2_ at both single-day and moving-average lag structures: single-day lags from the day and up to 6 days prior to the day of suicide (lag 0 to lag 6) and moving-average lags as the averages on 0–1, 0–3, and 0–6 days prior to the day of suicide (lag 0–1, lag 0–3, and lag 0–6). As public holidays may confound the association between suicide and air pollution (Lee et al., 2024; Tan et al., 2009), we included an indicator variable for public holidays in our single-lag models. As both suicide risk and the distribution of WBGT_max_ and air pollution demonstrate seasonal variability, we stratified the models by season. Both single-and two-exposure models were formulated to consider independent associations with and without adjustment for potential co-exposure confounding. The single-exposure model (e.g., a model of PM_2.5_) linked suicide with one environmental variable, whereas the two-exposure model (e.g., a model of PM_2.5_ + WBGT_max_) simultaneously linked suicide with two environmental variables. In the two-exposure model, the second exposure (e.g., WBGT_max_) was included as a linear term since previous studies within the U.S. have reported close to linear associations of air pollution and temperature with all-cause and suicide mortality (Di et al., 2017; Kim et al., 2019).

Three methods were used to examine the possible interactive effect of WBGT_max_ with each air pollutant on suicide. First, we examined the multiplicative interaction between WBGT_max_ and air pollutants, calculated by adding a product term (WBGT_max_ × air pollutant) to the two-exposure models to estimate the incremental change in suicide risk per 5-unit change in WBGT_max_ and 10-unit change in air pollutant. Second, we examined whether the combined effects of WBGT_max_ and air pollutants on suicide were more or less than additive by calculating the relative excess risk due to interaction (RERI) using the algorithm of VanderWeele and Knol (VanderWeele and Knol, 2014). To calculate RERI, we categorized WBGT_max_ and air pollutants into binary variables. Specifically, we used the median and 75th percentile of WBGT_max_ and air pollutants for the total period across all seasons as cut-off points to convert the continuous exposure measure into a binary variable, consistent with prior studies (Coker et al., 2025; Gong et al., 2024; Lee et al., 2018). Then, we created a new categorical variable by combining the binary cut-offs of the two exposures, yielding four categories: low-WBGT_max_ with low-PM_2.5_ or NO_2_ (reference group), low-WBGT_max_ with high-PM_2.5_ or NO_2_, high-WBGT_max_ with low-PM_2.5_ or NO_2_, and high-WBGT_max_ with high-PM_2.5_ or NO_2_. The RERI was computed using the following formula: RERI = OR11 – OR10 – OR01 + 1, where OR11 represents the risk in the level of high WBGT_max_ and high air pollution, OR_10_ represents the risk in the level of high WBGT_max_ and low air pollution, OR_01_ represents the risk in the level of low WBGT_max_ and high air pollution. An RERI of more than, equal to, or less than 0 suggest synergistic interactions (i.e., the combined effects of WBGT_max_ and PM_2.5_ or NO_2_ on suicide were greater than expected based on the estimated effects of each exposure alone), no additive interaction, or antagonistic interaction (i.e., less than additive joint effects), respectively. Third, we employed effect modification analysis to examine whether the associations of WBGT_max_ with suicide were different at different levels of air pollutants, indicating that there was an interaction between the two variables (Brambor et al., 2006). For this analysis, air pollutants were categorized into three groups (i.e., low, moderate, and high exposure levels) according to their tertiles (Tong et al., 2023). Conditional logistic regression models of WBGT_max_ and suicide were then stratified by tertile of air pollution exposure to measure effect modification. We tested the statistical significance of the observed difference in ORs across air pollution strata by calculating the 95% CI of the difference of coefficients between two strata of a potential effect modifier using the following formula (“difference test”): $\left(Q_{1}\;-\;Q_{2}\right)\;\pm \;1.96\sqrt{\left(\sigma_{1}^{2}\;+\;\sigma_{2}^{2}\right)}$, where *Q*_1_ and *Q*_2_ are the coefficients of the association for strata 1 and 2, and *σ*_1_ and *σ*_2_ are their respective standard errors (Schenker and Gentleman, 2001).

In addition, three-knot restricted cubic splines (Harrell, 2001) were fitted to assess the non-linearity and shape of the exposure–response curve between each environmental exposure and suicide, adjusting for all covariates included in the two-exposure models. The exposure–response curves were also plotted at different levels of air pollutants. The lag that yielded the largest difference in effect estimates between the high and low strata of the modifier was selected for exposure–response curves and sensitivity analyses.

### 2.5. Sensitivity analysis

Sensitivity analyses were conducted to assess the robustness of the main results. Specifically, we formulated two-pollutant models in which the relationship between suicide and the air pollutant of interest (e.g., NO_2_) was estimated by adding another pollutant (e.g., PM_2.5_) in the CLR model to assess the potential confounding of co-pollutants. To explore potential influencing factors in the interaction between WBGT_max_ and PM_2.5_ or NO_2_, we conducted subgroup analyses based on sex (female or male), age (>35 or ≤ 35 years old), and region (Salt Lake County or non-Salt Lake Counties). Salt Lake County was analyzed separately from the rest of Utah as it contains one-third of Utah’s population and serves as the hub for the state’s urban resources and facilities. We analyzed the seasonal effect of air pollution/WBGT_max_-suicide relationships by restricting data to the coldest (winter: December 21 to March 21) and warmest (summer: June 22 to September 21) parts of the year. In addition, we used alternative categorical definitions of low, moderate, and high air pollution exposures instead of tertiles based on < 25th, 25th–75th, and > 75th percentiles, respectively. Because holidays, such as Independence Day (July 4th), have been suggested to influence both suicide risk and air pollution levels (Beauchamp et al., 2014; Lee et al., 2024; Seidel and Birnbaum, 2015), we conducted a sensitivity analysis in which we excluded all case/control days on July 4th during the warm season and formulated single-pollutant (PM_2.5_ or NO_2_) unadjusted and adjusted CLR models on lags 0–1, 0–3, and 0–6. Finally, we computed E-values of two-exposure models to indicate the size of possible residual confounding (VanderWeele and Ding, 2017).

## 3. Results

### 3.1. Descriptive statistics

Table 1 describes the characteristics of suicide cases. Of the 7,551 suicide deaths from January 1, 2000 to December 31, 2016 in Utah, 77.1% (5,820) were male. The cases were distributed across Utah, with 40.9% (3,086) residing in Salt Lake County at their time of death. Most deaths happened on a weekday.

**Fig. 1.**
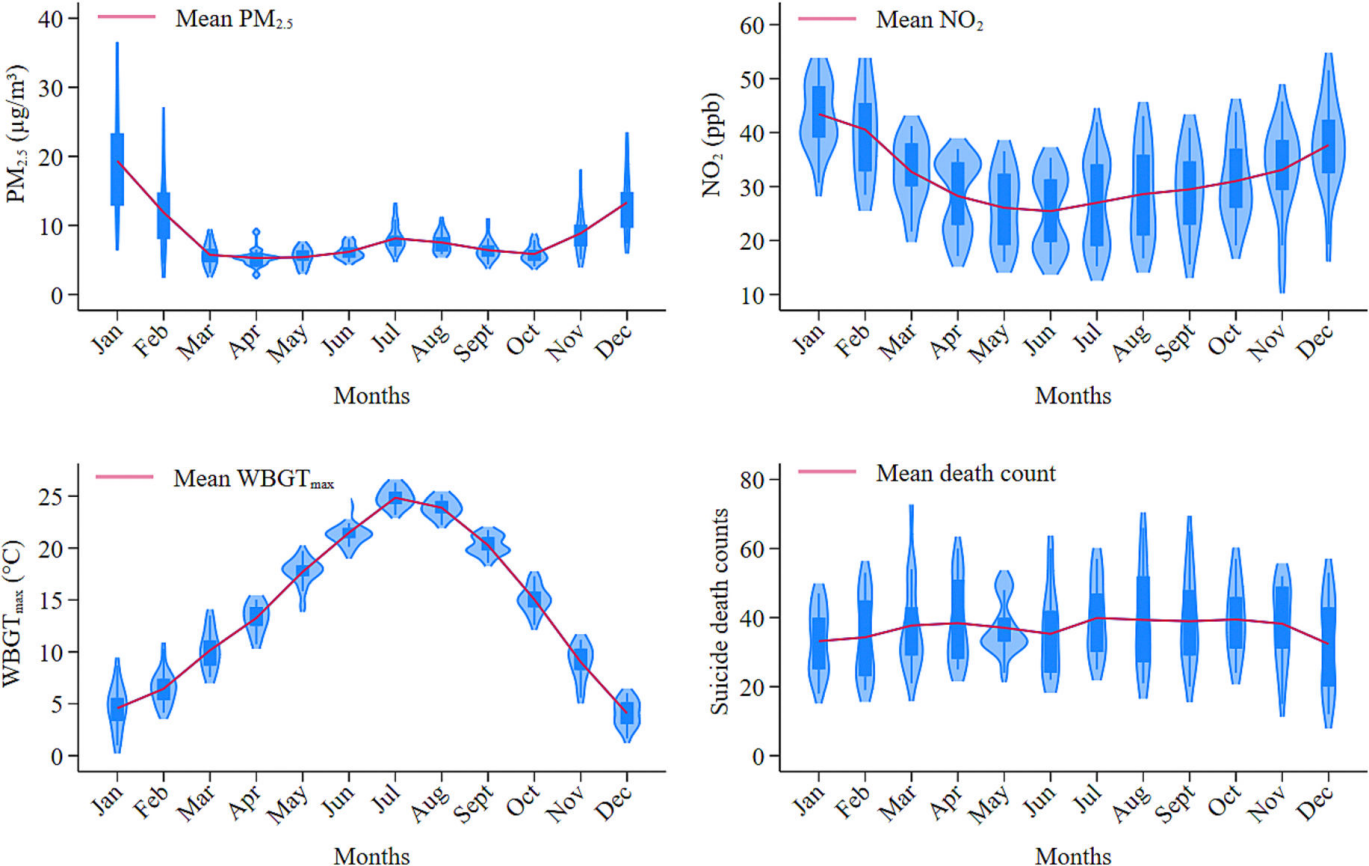
Monthly distributions of PM_2.5_, NO_2_, WBGT_max_, and suicide death counts in Utah, 2000–2016. Abbreviations: WBGT_max_, maximum wet bulb globe temperature; PM_2.5_, particulate matter with aerodynamic diameter ≤ 2.5 μm; NO_2_, nitrogen dioxide.

Table 2 summarizes the distributions of WBGT_max_ and air pollutants for the total period (both case and control periods) in Utah from 2000 to 2016. Across all seasons, the average daily WBGT_max_ was 14.6 °C, with daily ranges from −13.6 °C to 31.5 °C. The average values of air pollution concentrations were 8.3 μg/m^3^ (range: 0.2–171.2 μg/m^3^) for daily mean 24-hour PM_2.5_ and 30.0 ppb (range: 0.4–162.3 ppb) for daily 1-hour maximum NO_2_. The mean WBGT_max_ of the cold and warm seasons were 9.1 °C and 19.8 °C, respectively. PM_2.5_ and NO_2_ concentrations were higher in the cold season or at low WBGT_max_. There were no obvious differences in the average WBGT_max_, PM_2.5,_ and NO_2_ between case and control periods (Table S1). PM_2.5_ and NO_2_ showed higher average values from December to February than in other months, while higher average values of WBGT_max_ were observed from June to August (Fig. 1). In addition, we observed a higher mean number of suicides in the warmer months (July to September), with suicides peaking in July. Furthermore, correlations of WBGT_max_ with PM_2.5_ and NO_2_ were weak and negative (Pearson’s correlation coefficient (*r*) ranged from −0.19 to −0.27) and positive correlations between PM_2.5_ and NO_2_ were moderate (*r* = 0.50 and 0.57, respectively) for all seasons and the cold season (Fig. S1).

### 3.2. Independent effects of WBGT_max_ and air pollutants on suicide mortality

Table 3 shows the associations of WBGT_max_ and NO_2_ with suicide mortality across the entire sample and by season from the single- and two-exposure models on moving-average lag days; the results on the - single-day lags are reported in the supplementary file (Table S2). Across all seasons, the single-exposure models indicated that a 5 °C increase in WBGT_max_ was significantly associated with suicide on single-day lags 0 and 2 and moving-average lag days on lag 0–1 and lag 0–3, with the strongest associations on lag 0–3 days (OR = 1.05, 95% CI: 1.01, 1.10). In the warm season, suicide was significantly associated with WBGT_max_ on all lag structures, with the ORs ranging from 1.08 (95% CI: 1.02, 1.15) to 1.20 (95% CI: 1.10, 1.30) per 5 °C increase, depending on the lag periods. During the cold season, no relationship was detected between WBGT_max_ and suicide. The relationships observed between WBGT_max_ and suicide in the single-exposure models were consistent with the two-exposure models when air pollutants were included (Tables 3 and Table S2).

Across all seasons, NO_2_ (per 10 ppb increase) was associated with an increase in suicide risk only on lag 2 according to the single-and two-exposure models (Table S2). Following stratification by season, NO_2_ was associated with an increased suicide risk during the cold season on all moving-average lags and most single-day lags based on single- and two-exposure models, with the greatest OR of NO_2_ observed on lag 0–6 in the two-exposure model (OR = 1.14; 95% CI: 1.07, 1.22) (Tables 3 and Table S2). In the warm season, the single-exposure models indicated no statistically significant association of NO_2_ with suicide for most lag periods. However, NO_2_ was found to be inversely associated with suicide during the warm season in the two-exposure models. The findings across all seasons and during the warm season for PM_2.5_ were similar to those for NO_2_ in most lag periods (Table S2). In the cold season, a positive relationship between PM_2.5_ and suicide was significant on lag 3 for the single-exposure model and lag 4 for the two-exposure model. A test for differences in effect estimates found significant differences (*P* < 0.05) in the associations of WBGT_max_, PM_2.5_, and NO_2_ with suicide between the warm and cold seasons (Tables 3 and Table S2).

### 3.3. Interactive effects of WBGT_max_ and air pollutants on suicide mortality

Table 4 displays results of the multiplicative interactions between WBGT_max_ and PM_2.5_ or NO_2_ on suicide. Across all seasons, WBGT_max_ and NO_2_ were not found to interact on multiplicative scales on any moving-average or single-day lags. However, following stratification by season, we found that the multiplicative interactions between WBGT_max_ and NO_2_ for most moving-average and single-day lags were positive and statistically significant in both the cold and warm seasons, indicating the presence of a synergistic interaction. Stronger synergistic effects were observed on lag 0–6 in the warm season, with a 5 °C increase in WBGT_max_ and 10 ppb increase in NO_2_ associated with an OR of suicide of 1.16 (95% CI: 1.09, 1.23). The multiplicative interactions between WBGT_max_ and PM_2.5_ were positive and statistically significant on lag 3 during the warm season (OR = 1.14, 95% CI: 1.03, 1.26), while no consistent interactions were observed across other lags (Table S3). Table 5 presents results from the analysis of additive interaction and shows that WBGT_max_ and NO_2_ interacted synergistically (RERI > 0) to increase suicide odds. Statistical significance was found for the 50th percentile of WBGT_max_ and the 50th and 75th percentiles of NO_2_ in the warm season. The RERIs ranged from 0.26 (95% CI: 0.04, 0.49) to 0.37 (95% CI: 0.12, 0.63), with the strongest synergistic effects observed on lag 0–6. Additive interaction for suicide was also detected for PM_2.5_ and WBGT_max_ in the warm season when WBGT_max_ and PM_2.5_ were defined by the 50th and 75th percentiles, respectively (Table S4). However, significant antagonistic effects (RERI < 0) of WBGT_max_ and PM_2.5_ exposures on suicide were detected across all seasons.

**Fig. 2.**
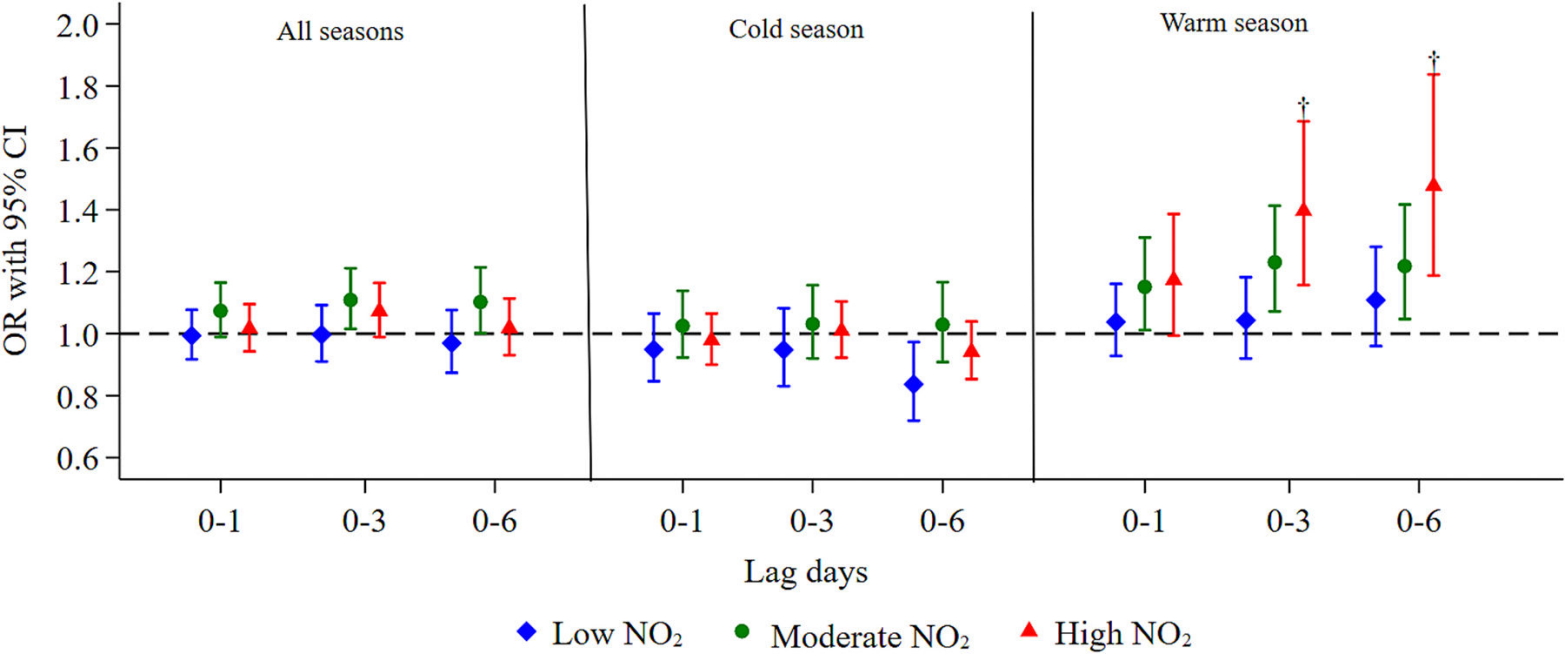
Associations between a 5 °C increase in WBGT_max_ and suicide deaths by three levels [low (tertile 1), moderate (tertile 2), and high (tertile 3)] of NO_2_ for moving-average lag day models. Error bars represent 95% CI for estimated ORs. Abbreviations: WBGT_max_, maximum wet bulb globe temperature; NO_2_, nitrogen dioxide; OR, odds ratio; CI, confidence interval. ^†^ Differences between the two coefficients of the subgroup (high and low NO_2_) are statistically significant at *P* < 0.05.

Furthermore, the effect modification analyses in the warm season showed stronger associations between WBGT_max_ and suicide at high NO_2_ levels (Fig. 2 and Table S5). We found a monotonic trend in the heat-related suicide risk across different tertiles of NO_2_, with estimates highest on high NO_2_ days and lowest on days with low NO_2_ levels. The effect estimates of WBGT_max_ between high and low NO_2_ levels were statistically different on single-day lags 5 and 6 and moving-average lags 0–3 and 0–6, with the biggest difference in the estimates occurring on lag 0–6 (Tables S5 and S6). For example, during the warm season on moving-average lag 0–6, the OR for suicide per 5 °C increase in WBGT_max_ was 1.48 (95% CI: 1.19, 1.84) on high NO_2_ days and 1.11 (95% CI: 0.96, 1.28) on low NO_2_ days, with an OR difference of 0.29 (95% CI: 0.03, 0.55) (Table S5). The effect modification analyses for PM_2.5_ showed stronger associations between WBGT_max_ and suicide at high PM_2.5_ levels on lag 2 in the warm season, but this relationship was not observed in other lag periods (Tables S5 and S6).

### 3.4. Exposure-response curves

**Fig. 3.**
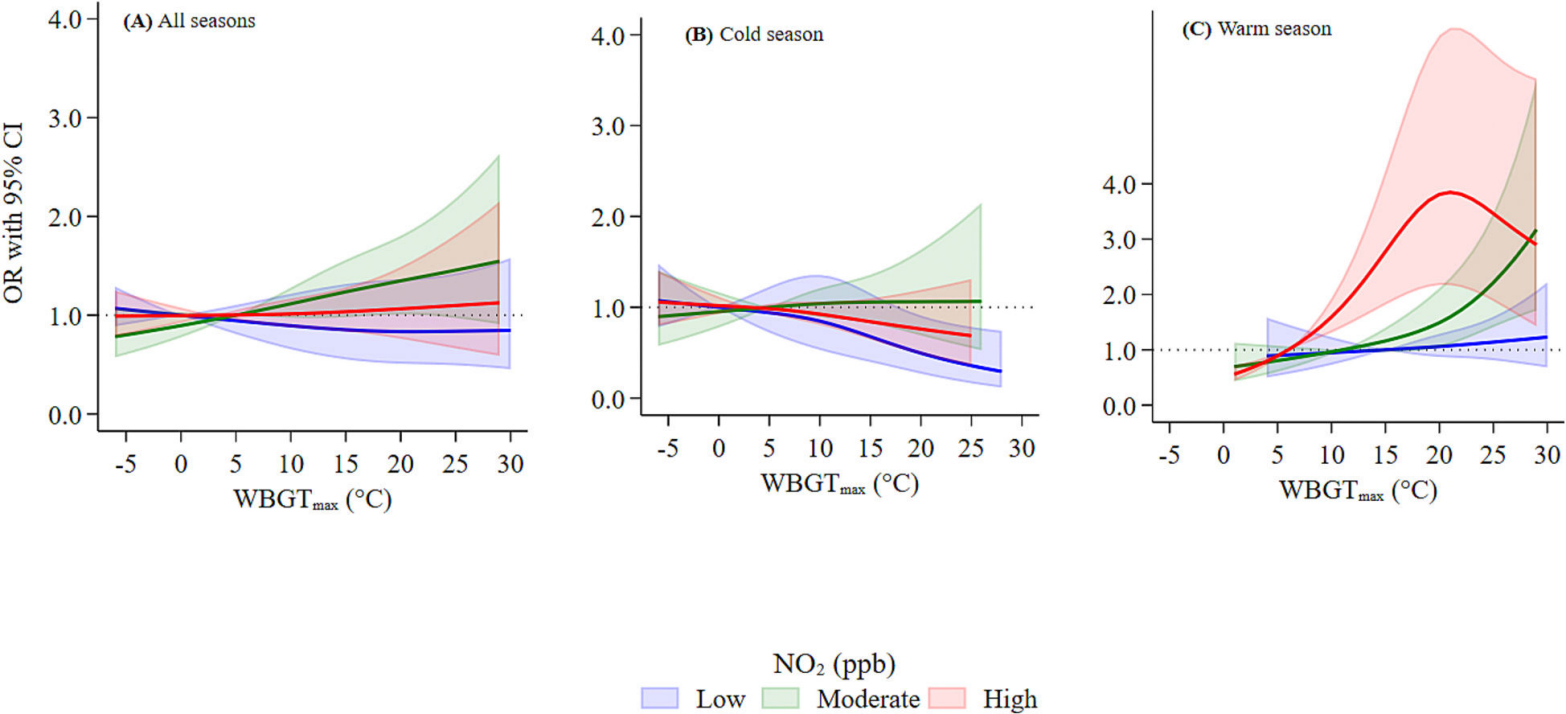
Exposure-response curves of the association between WBGT_max_ and suicide mortality under different tertiles of NO_2_ during the moving-average lag of 0–6 for all seasons (A), cold season (B), and warm season (C). The solid lines with shaded areas represent the ORs and their 95% CIs. This curve was calculated using the restricted cubic splines for WBGT_max_ concentrations with knots at the 10th, 50th, and 90th percentiles. The blue solid line with the blue shaded area is for low (tertile 1) NO_2_ levels; the green solid line with the green shaded area is for moderate (tertile 2) NO_2_ levels; and the red solid line with the red shaded area is for high (tertile 3) NO_2_ levels. Abbreviations: WBGT_max_, maximum wet bulb globe temperature; NO_2_, nitrogen dioxide; OR, odds ratio; CI, confidence interval; ppb, parts per billion. (For interpretation of the references to colour in this figure legend, the reader is referred to the web version of this article.)

The exposure–response relationship curves for WBGT_max_ across the entire year and by season were approximately linear: the slope of the curve for the warm season was steeper, while the curve for cold season was flatter (Fig. S2). We also found a monotonic trend in the WBGT_max_-suicide association across different tertiles of NO_2_ during the warm season (Fig. 3), which aligns with findings from our moderation analysis (Fig. 2). We found differences in the exposure–response curves for PM_2.5_ and NO_2_ exposures and suicide during different seasons (Fig. S2). The curves depicted increased suicide risk associated with higher NO_2_ during the cold season, whereas PM_2.5_ and NO_2_ showed the opposite trends during the warm season.

### 3.5. Sensitivity analysis

Results of the two-pollutant models for the air pollution–suicide associations showed the attenuation of PM_2.5_ ORs after adjustment for NO_2_ during the cold season (Fig. S3A), whereas ORs for NO_2_ remained stable after adjustment for PM_2.5_ (Fig. S3B). When the analyses were stratified by sex and age group, we found that the ORs of suicide for NO_2_ exposure during the cold season were higher among males and persons aged 36–64 years (Tables S7 and S8). The ORs for heat-related suicide was higher among females and persons aged 36–64 years. Furthermore, positive multiplicative interactions between NO_2_ and WBGT_max_ on suicide were measured during the warm season for both males and females, as well as for those aged ≤ 35 and 36–64 years. In our regional analysis, we found significant positive associations between WBGT_max_ and suicide in Salt Lake (SL) and non-SL counties during the warm season, whereas NO_2_ was positively associated with suicide in SL county only (Table S9). In addition, the multiplicative interaction between warm-season WBGT_max_ and NO_2_ was positive and statistically significant in SL and non-SL counties, with stronger interactive effects observed in SL county. When we restricted our analysis to the summer season, associations between WBGT_max_ and suicide were enhanced (Table S10). Additionally, in the winter, ORs for NO_2_ were robust and slightly enhanced, while PM_2.5_ became significantly associated with suicide in the two-exposure model. Using percentile cut-offs for air pollution categories instead of tertiles did not materially change the effect modification analyses (Table S11). Estimates obtained from the unadjusted and adjusted models excluding case and control data on Independence Day were consistent with estimates from the main analysis (Table S12). Finally, we did not find strong evidence for unmeasured confounding based on E-value estimation (Table S13).

## 4. Discussion

In this study, we used a case-crossover design to investigate the independent and interactive effects of ambient WBGT_max_ and air pollutants on suicide mortality in Utah, USA. We observed that exposure to higher WBGT_max_ across the entire population and during the warm season and to NO_2_ in the cold season were independently associated with increased risks of suicide. Further, we observed linear exposure–response relationships between WBGT_max_ and NO_2_ with suicide. WBGT_max_ was found to interact synergistically with PM_2.5_ and NO_2_ to influence suicide risk during the warm season, with the contribution of heat stress to suicide most pronounced on days with high NO_2_ concentrations.

### 4.1. Independent effects of WBGT_max_ and air pollutants

Our study found an association between a 5 °C increase in WBGT_max_ and suicide across the entire year, with the amplification of this relationship during the warm season. Similar findings have been reported in prior work for ambient temperature other than the WBGT (Frangione et al., 2022; Hertzog et al., 2024; Rahman et al., 2023; Schneider et al., 2020; Villeneuve et al., 2023). A meta-analysis of 10 case-crossover studies found a link between ambient temperature (considering lags up to 5 days) and suicide (Frangione et al., 2022). A time-stratified case-crossover study of more than 50,000 suicides over a 14-year period in Canada found that an interquartile range increase in daily temperature (9.6 °C) on the day of death was associated with a 10.1% (95% CI: 9.0%, 11.2%) increase in the odds of suicide, with the strongest association measured in the warm season (Villeneuve et al., 2023). In the U.S., a time-stratified case-crossover study in California including 24,387 records of suicide deaths revealed a significant association between daily temperature and suicide (Rahman et al., 2023). Our study advances prior work using WBGT instead of ambient temperature. Wet bulb globe temperature is considered a more accurate metric of heat and perceived temperature than ambient temperature as it accounts for the combined effects of air temperature, humidity, wind speed, and solar radiation (Wodzicki et al., 2024). Hence, we expect that our use of WBGT_max_ yields more accurate measures for predicting the impact of heat on future suicide risk than previous work.

We found NO_2_, which is often used as a proxy for traffic-related pollution, to be primarily associated with increased odds of suicide during the cold season. This is consistent with expectations based on seasonal meteorological phenomenon in Utah. NO_2_ concentrations tend to accumulate in Utah during the winter due to the regular occurrence of air inversion events that result from inverted temperature layers, which trap air pollution from vehicle and commercial combustion and residential heating in valley floors (Beard et al., 2012; Bishop et al., 2022). Air pollution exposure during the winter and wintertime air inversion events in Utah have been associated with other negative health outcomes, including increased rates of emergency department visits for asthma and acute coronary syndromes (Beard et al., 2012; Horne et al., 2023; Silva et al., 2007). As for suicide, prior Utah-based research reported a year-round association between short-term exposure to higher NO_2_ and suicide among decedents residing in Salt Lake County from 2000 to 2010 (Bakian et al., 2015). In the current study, we did not observe a year-round association between short-term NO_2_ and suicide. However, in a sensitivity analysis in which the study population was restricted to Salt Lake County, average NO_2_ concentrations were higher (34 ppb on case days) than the average NO_2_ concentrations for the remainder of the state (28 ppb on case days), a positive association between short-term NO_2_ and suicide was observed. Consistent with prior studies (Faustini et al., 2014), despite the correlation between NO_2_ and PM_2.5_ due to both originating from common sources including fossil fuel combustion (Mukerjee et al., 2019), the relationship between NO_2_ and suicide remained robust after adjustment with PM_2.5_, suggesting independent effects of NO_2_.

PM_2.5_ exposure was only associated with suicide risk in the winter, in contrast to prior research conducted in Salt Lake County, Utah, which found a year-round association between PM_2.5_ and suicide (Bakian et al., 2015). Like NO_2_, higher concentrations of PM_2.5_ in Utah have historically been measured during wintertime air inversion events. More recently, however, peak PM_2.5_ concentrations in Utah have occurred during the summer because of widespread wildfire events (Horne et al., 2024). The recentness of wildfires means that the current study, which covers suicide deaths in Utah from 2000 to 2016, did not quantify the relationship between wildfire-based PM_2.5_ and suicide. Future research will want to use more contemporary data to investigate and compare the potential differential contributions of PM_2.5_ originating from wildfire events versus wintertime inversions to suicide. This will become more crucial as climate change is projected to make wildfires more frequent and intense.

The current study measured changes in suicide risk across a six-day exposure window preceding death with the intention to identify critical windows of exposure. The identification of specific exposure windows may yield new insights into potential biological mechanisms underlying suicide. We found that the association between WBGT_max_ and suicide generally increased across increasing lags with the strongest effects of WBGT_max_ measured on lag 0–6 during the warm season. Similarly, for NO_2_, the strongest association with suicide was measured on lag 0–6 during the cold season. However, our effect estimates for WBGT_max_ and NO_2_ did not plateau within the six days preceding suicide, suggesting that the critical exposure window may exceed six days. Currently, no consensus exists across suicide studies on what the critical exposure windows are for short-term heat and air pollution exposures (Casas et al., 2022; Davoudi et al., 2021; Frangione et al., 2022), suggesting the need for further research in additional populations at varied geographies, latency periods, and study designs.

In the present study, we observed a linear exposure–response curve for the relationship between WBGT_max_ and suicide, with the relationship progressively increasing as WBGT_max_ increased in the warm season. Similar findings have been reported in the exposure–response relationships for heat metrics other than WBGT in Western countries including the U.S. (Kim et al., 2019; Lehmann et al., 2022; Rahman et al., 2023), in contrast to northeast Asian countries (Japan, South Korea, and Taiwan), where inverted J-shaped curves have been reported (Kim et al., 2019). Although the exposure–response relationship between WBGT and suicide has not been reported, previous studies revealed a linear relationship between WBGT and heat-related illnesses in Japan (Nakamura et al., 2022; Ueno et al., 2021), which is consistent with our findings. Similarly, for PM_2.5_ and NO_2_, the exposure–response curves were linear without a clear threshold, which is consistent with prior research (Lian et al., 2024). Interestingly, we identified seasonal differences in the slopes of the curves; the slope for the cold season was positive, while the slope for the warm season was negative. This indicates that climatic factors, such as temperature and humidity, may modify the concentration, chemical composition, and dispersion of pollutants (Shen et al., 2021), leading to seasonal variations in the risk of suicide linked to air pollutants.

Our finding of inverse associations of PM_2.5_ and NO_2_ with suicide during the warm season was unexpected and merits careful interpretation. One possible explanation for this association is the substantially lower pollutant concentrations experienced in Utah during the warm season, particularly relative to winter. In our study, the mean warm-season concentrations of PM_2.5_ (6.4 μg/m^3^) and NO_2_ (26.0 ppb) were markedly lower than those observed during the cold season (PM_2.5_ = 10.2 μg/m^3^ and NO_2_ = 34.4 ppb). Such lower warm-season concentrations may reduce exposure contrast and shift pollutant composition (Boogaard et al., 2024; Hand et al., 2024), potentially weakening or qualitatively altering exposure–response relationships through threshold effects, increased exposure misclassification, and residual confounding by correlated seasonal factors. In addition, the composition of fine particulate matter and its toxicity likely vary seasonally (Jovanovic et al., 2020), potentially resulting in qualitatively different health effects across seasons. Similar inverse relationships have been observed in a California-based time-series study, where exposure to NO_2_ (mean:10.7 ppb) was associated with a decreased risk of emergency department visits for mental disorders, including suicide/self-harm (Thilakaratne et al., 2020). In contrast, other studies that examined lower air pollutant concentrations during the warm season demonstrated positive (Villeneuve et al., 2023) or null (Rahman et al., 2023) associations between PM_2.5_ and NO_2_ with suicide mortality. Heterogeneous findings among studies may suggest a role for additional factors that correlate with pollution concentration levels and vary regionally, such as meteorological, housing, and human activity patterns.

### 4.2. Interactions between WBGT_max_ and air pollutants

Understanding the combined effects of WBGT and air pollution is crucial for informing accurate risk assessments, yet such information has largely been lacking for suicide. We found significant synergistic effects of PM_2.5_ and NO_2_ and WBGT_max_ on suicide during the warm season. Synergistic effects between short-term exposure to heat and air pollutants have been previously reported for all-cause mortality (Lee et al., 2019; Scortichini et al., 2018; Stafoggia et al., 2023). A multi-country study that included the U.S. found evidence of multiplicative interactions between short-term exposure to ambient temperature and PM_2.5_ and NO_2_ on all-cause mortality during the warm season (Stafoggia et al., 2023). Another multi-country study of 16 Northeast Asian cities reported additive interactions between short-term exposures to high temperature and NO_2_ on all-cause mortality (Lee et al., 2019). The direction of associations in these all-cause mortality studies is aligned with our findings for the interaction of WBGT_max_ with PM_2.5_ and NO_2_ during the warm season. In contrast, we found that the joint effects of PM_2.5_ and WBGT_max_ on suicide were less than additive (RERIs < 0) across the entire year. The composition of PM_2.5_ is temperature sensitive, with specific components increasing with higher temperatures and others decreasing (Yin et al., 2025). The antagonistic interaction between WBGT_max_ and PM_2.5_ in our study may be due to higher distribution ranges of temperature and temperature-dependent composition of PM_2.5_ (Castro et al., 2025; Xu et al., 2023; Yin et al., 2025). However, further studies are needed to explicitly test the interactions between WBGT and the chemical composition of PM_2.5_ on suicide.

Effect modification analysis by NO_2_ revealed that association between WBGT_max_ and suicide was stronger on days with higher NO_2_ levels during the warm season, suggesting that reducing the concentrations of NO_2_ might yield greater benefits for reducing suicide risk than intervening on heat exposure alone. Air pollutants have been previously shown to modify the effect of ambient temperature on suicide mortality (Villeneuve et al., 2023) and non-suicide mortality outcomes (Rai et al., 2023; Zhang et al., 2024). For example, a multi-country study found that the effects of temperature on cardiorespiratory mortality risk were modified by elevated levels of NO_2_ during warmer months and substantial effect modifications were seen in Switzerland, where the risk for heat-related mortality increased from 18.6% to 43.5% on days with low (5th percentile) and high (95th percentile) NO_2_ levels, respectively (Rai et al., 2023), which is comparable with our estimates.

### 4.3. Potential biological mechanisms

Several mechanisms have been proposed to underlie the synergistic effects of short-term exposure to heat and air pollutants on suicide risk. First, heat exposure can activate thermoregulatory responses, including increased ventilation rate and lung volumes, which in turn can directly or indirectly influence the entry of harmful substances into the body and increase the total intake of air pollution (De Sario et al., 2013; Gordon, 2003). In addition, rising body temperature during heat exposure can alter physiological responses to chemicals and intensify the harmful effects of air pollution (Gordon et al., 2014). Second, heat and air pollution share common biological pathways, including oxidative stress and neuroinflammation (Catapano et al., 2024; Lohmus, 2018), which are not only directly associated with suicide (García-Gutiérrez et al., 2025; Koweszko et al., 2020), but may also indirectly increase the risk of suicide due to their association with exacerbating underlying mental health conditions (Qiu et al., 2022). In addition, high temperature and air pollution may interfere with neurotransmitter balance in the brain, affecting serotonin and dopamine levels, which are crucial chemicals in the regulation of mood and behavior (Seo et al., 2008). A deficiency of these chemicals, particularly serotonin, is associated with increased aggression and suicide (Maes et al., 1995; da Cunha-Bang and Knudsen, 2021). Serotonin levels tend to decrease with higher temperature and air pollution levels (Tiihonen et al., 2017; Fossier et al., 1999).

### 4.4. Strengths and limitations

Study strengths include the assessment of exposure with high spatial and temporal resolution, the use of a case-crossover design to minimize potential seasonal and individual-level confounders, and the examination of multiplicative and additive interactions between WBGT_max_ and air pollutants. We also identified a dose–response relationship between air pollution and WBGT_max_ on suicide mortality using non-linear modeling, providing deeper insight into interaction analysis and strengthening the validity of our findings. Furthermore, this study fills the gap of previous research that mainly focused on the interaction between air pollution and temperature but ignored variability in these relationships by season.

This study is not without limitations. First, information on the mobility of the study population is lacking; exposure assignments are based on the residential locations indicated on death certificates. Residential mobility, such as moving from areas with low air pollution to high air pollution or from colder to warmer regions, could influence the short-term associations observed in our study. However, while such mobility could modify the strength of the associations, it is unlikely to confound them, since the long-term exposure history remains consistent between case and control days (Qiu et al., 2022). Second, exposure assessment relied on residential addresses reported on death certificates rather than the location of death. For some individuals, the location of death may have been different from their residential address, potentially introducing measurement errors. However, we anticipate that these errors were minimal due to the limited differences in exposure measures over short spatial ranges. Third, exposure assessments were based on nationwide models applied to local areas, meaning we lacked precise information on the uncertainty of exposure estimates across different areas in Utah, particularly in remote regions of Utah that lacked monitoring stations. Likewise, ambient exposures were used as surrogates for the actual exposures, which may have primarily been in indoor environments where heating, ventilation, air conditioning, filtration, and indoor sources may influence heat stress and air pollutant levels. Nevertheless, high exposure measurement errors would bias the effect estimates towards the null, thereby underestimating the true associations. Fourth, Utah is a geographically diverse state with a wide range of climates, from hot and dry summers to cold and snowy winters, and with a sociodemographically diverse population, which improves the generalizability of our findings to other regions of the U.S. Nonetheless, to establish the extent to which these findings are broadly generalizable, confirmation in settings with different climatic conditions, demographic structures, and baseline air pollution and suicide profiles is needed. Finally, the most recent data are nearly nine years old, leaving uncertainty regarding whether the observed exposures and outcomes remain consistent with more current data that includes increased PM_2.5_ from wildfires in the warm season.

## 5. Conclusions

Our study provides evidence that short-term exposure to WBGT_max_ was independently associated with suicide mortality, with enhanced effects observed during the warm season. The interactive effects of WBGT_max_ with PM_2.5_ and NO_2_ on suicide risk during the warm season were synergistic; the risks of heat-related suicide were amplified on days with higher NO_2_ levels. This study adds new evidence to the current understanding of the interactive effects of air pollution and weather variables on suicide and suggests taking steps to reduce exposure to heat during the warm season, but to pay special attention when the air quality deteriorates on hot days.

## Supplementary Material

supplementary file

## Figures and Tables

**Table 1:** General characteristics of suicide decedents in Utah, the U.S., between 2000 and 2016.

Characteristics	Total (N = 7,551)	
	N	%

Age group (years)		
≤ 35	3,073	40.7
36–64	3,699	49.0
≥65	779	10.3
Sex		
Male	5,820	77.1
Female	1,686	22.3
Missing	45	0.6
Season		
Cold	3,663	48.5
Warm	3,888	51.5
Region		
Salt Lake County	3,086	40.9
Non-Salt Lake Counties	4,420	58.5
Missing	45	0.6
Day of week		
Sunday	1,055	14.0
Monday	1,133	15.0
Tuesday	1,167	15.4
Wednesday	1,069	14.2
Thursday	1,078	14.3
Friday	1,087	14.4
Saturday	962	12.7
Holiday	235	3.1

**Table 2:** Summary statistics for air pollution and WBGT_max_ by season and WBGT_max_ group (total period).

Exposure	Mean (SD)	Percentiles			Min	Max
		P25	P50	P75		

*WBGT_max_ (°C)*						
All seasons	14.62 (8.03)	8.26	15.33	21.59	−13.59	31.45
Cold season	9.12 (6.40)	4.43	8.64	13.68	−13.59	28.52
Warm season	19.76 (5.63)	16.30	21.00	24.05	−3.02	31.45
Low WBGT_max_	5.22 (3.70)	2.89	5.88	8.26	−13.59	10.41
Moderate WBGT_max_	15.17 (2,71)	12.75	15.33	17.55	10.41	19.64
High WBGT_max_	23.47 (2.31)	21.59	23.29	25.15	19.64	31.35
*PM_2.5_ (μg/m^3^*)						
All seasons	8.25 (8.69)	4.06	5.86	8.70	0.23	171.20
Cold season	10.24 (11.62)	3.87	6.14	11.50	0.23	171.20
Warm season	6.38 (3.55)	4.21	5.71	7.63	0.42	52.80
Low WBGT_max_	11.31 (13.10)	3.56	6.19	13.66	0.23	171.20
Moderate WBGT_max_	6.20 (5.21)	3.63	4.98	6.95	0.38	118.04
High WBGT_max_	7.23 (3.61)	5.06	6.47	8.36	1.08	52.80
*NO_2_ (ppb)*						
All seasons	30.04 (14.45)	18.98	29.72	39.98	0.38	162.31
Cold season	34.38 (14.87)	24.04	34.88	43.81	0.38	162.31
Warm season	25.98 (12.77)	15.92	25.21	34.92	0.50	118.14
Low WBGT_max_	35.34 (15.78)	24.25	36.06	45.21	0.38	162.31
Moderate WBGT_max_	28.25 (13.09)	17.80	28.54	37.88	0.84	107.64
High WBGT_max_	26.52 (12.72)	16.78	25.63	35.15	0.50	118.14

Abbreviations: WBGT_max_, maximum wet bulb globe temperature; PM_2.5_, particulate matter with aerodynamic diameter ≤ 2.5 μm; NO_2_, nitrogen dioxide; SD, standard deviation; P, percentile; ppb, parts per billion.

**Table 3:** Odds ratios of suicide mortality associated with each 5 °C increase in WBGT_max_ and 10 unit increase in PM_2.5_ and NO_2_ from single- and two-exposure models on moving-average lag days.

Models	Exposure	Season	Lag 0–1	Lag 0–3	Lag 0–6
			OR (95% CI)	OR (95% CI)	OR (95% CI)

Single-exposure model^a^	PM_2.5_	All seasons	0.99 (0.95, 1.03)	1.01 (0.97, 1.06)	1.02 (0.97, 1.07)
		Cold season	1.01 (0.97, 1.05)	1.03 (0.98, 1.07)	1.03 (0.98, 1.09)
		Warm season	**0.84 (0.73, 0.96)**	**0.84 (0.72, 0.99)**	0.86 (0.72, 1.03)
	NO_2_	All seasons	1.02 (0.98, 1.06)	1.04 (0.99, 1.09)	1.05 (0.99, 1.11)
		Cold season	**1.06 (1.01, 1.11)**	**1.10 (1.04, 1.16)**	**1.13 (1.06, 1.21)**
		Warm season	0.95 (0.89, 1.01)	0.93 (0.86, 1.01)	0.89 (0.81, 0.98)
	WBGT_max_	All seasons	**1.04 (1.00, 1.08)**	**1.05 (1.01, 1.10)**	1.04 (0.99, 1.09)
		Cold season	1.00 (0.95, 1.05)	1.01 (0.95, 1.07)	0.96 (0.90, 1.02)
		Warm season	**1.11 (1.04, 1.19)**	**1.14 (1.06, 1.22)**	**1.20 (1.10, 1.30)**
Two-exposure model (PM_2.5_ and WBGT_max_)^b^	PM_2.5_	All seasons	0.98 (0.95, 1.02)	1.00 (0.96, 1.05)	1.01 (0.96, 1.07)
	WBGT_max_	All seasons	**1.04 (1.00, 1.08)**	**1.05 (1.01, 1.10)**	1.04 (0.98, 1.09)
	PM_2.5_	Cold season	1.01 (0.97, 1.05)	1.03 (0.98, 1.07)	1.04 (0.99, 1.09)
	WBGT_max_	Cold season	0.99 (0.94, 1.05)	1.00 (0.95, 1.06)	0.95 (0.89, 1.02)
	PM_2.5_	Warm season	**0.77 (0.67, 0.89)** ^†^	**0.76 (0.65, 0.90)** ^†^	**0.76 (0.63, 0.92)** ^†^
	WBGT_max_	Warm season	**1.15 (1.08, 1.23)** ^†^	**1.18 (1.09, 1.27)** ^†^	**1.24 (1.13, 1.35)** ^†^
Two-exposure model (NO_2_ and WBGT_max_)^c^	NO_2_	All seasons	1.01 (0.97, 1.05)	1.03 (0.98, 1.08)	1.04 (0.99, 1.10)
	WBGT_max_	All seasons	1.04 (0.99, 1.08)	**1.05 (1.00, 1.10)**	1.03 (0.98, 1.09)
	NO_2_	Cold season	**1.06 (1.01, 1.12)**	**1.10 (1.04, 1.17)**	**1.14 (1.07, 1.22)**
	WBGT_max_	Cold season	0.98 (0.93, 1.03)	0.99 (0.94, 1.05)	0.95 (0.89, 1.01)
	NO_2_	Warm season	**0.92 (0.86, 0.98)** ^†^	**0.90 (0.83, 0.98)** ^†^	**0.86 (0.78, 0.95)** ^†^
	WBGT_max_	Warm season	**1.14 (1.06, 1.22)** ^†^	**1.16 (1.08, 1.25)** ^†^	**1.22 (1.12, 1.33)** ^†^

Abbreviations: WBGT_max_, maximum wet bulb globe temperature; PM_2.5_, particulate matter with aerodynamic diameter ≤ 2.5 μm; NO_2_, nitrogen dioxide; OR, odds ratio; CI, confidence interval.

Statistically significant (*P* < 0.05) associations are marked in bold.

a This model included only WBGT_max_, PM_2.5_, or NO_2_ to test the main effect of each 5 °C increase in WBGT_max_ or 10 unit increase in PM_2.5_ or NO_2_ on suicide mortality.

b This model included WBGT_max_ and PM_2.5_ simultaneously to test the main effects of each 5 °C increase in WBGT_max_ and each 10 μg/m^3^ increase in PM_2.5_ on suicide mortality.

c This model included WBGT_max_ and NO_2_ simultaneously to test the main effects of each 5 °C increase in WBGT_max_ and each 10 ppb increase in NO_2_ on suicide mortality.

† Differences between the two coefficients of subgroup analysis (cold and warm seasons) are statistically significant at *P* < 0.05.

**Table 4:** Multiplicative interactions between WBGT_max_ and air pollution for suicide mortality on moving-average lag days.

Exposure	Lag days	All seasons	Cold season	Warm season
		OR (95% CI)^c^	OR (95% CI)^c^	OR (95% CI)^c^

PM_2.5_ and WBGT_max_^a^	0–1	0.97 (0.94, 1.00)	1.02 (0.97, 1.07)	1.01 (0.89, 1.14)
	0–3	0.98 (0.94, 1.01)	1.02 (0.97, 1.08)	1.12 (0.97, 1.29)
	0–6	0.98 (0.93, 1.02)	1.03 (0.97, 1.10)	1.13 (0.96, 1.33)
NO_2_ and WBGT_max_^b^	0–1	1.01 (0.99, 1.03)	**1.04 (1.01, 1.08)**	**1.05 (1.01, 1.09)**
	0–3	1.01 (0.99, 1.04)	**1.06 (1.02, 1.10)**	**1.10 (1.05, 1.16)**
	0–6	1.01 (0.99, 1.04)	**1.07 (1.02, 1.11)**	**1.16 (1.09, 1.23)**

Abbreviations: WBGT_max_, maximum wet bulb globe temperature; PM_2.5_, particulate matter with aerodynamic diameter ≤ 2.5 μm; NO_2_, nitrogen dioxide; OR, odds ratio; CI, confidence interval.

Statistically significant (*P* < 0.05) associations are marked in bold.

a Interaction term (WBGT_max_ × PM_2.5_) was added into the two-exposure model to test the interaction between each 5 °C increase in WBGT_max_ and each 10 μg/m^3^ increase in PM_2.5_ on suicide mortality.

b Interaction term (WBGT_max_ × NO_2_) was added into the two-exposure model to test the interaction between each 5 °C increase in WBGT_max_ and each 10 ppb increase in NO_2_ on suicide mortality.

c OR and 95% CI for the interaction term. A significant interaction term indicates the existence of multiplicative interaction.

**Table 5:** Relative excess risk due to interaction (RERI) of WBGT_max_ and NO_2_ on suicide mortality on moving-average lag days.

WBGT_max_^a^	NO_2_^b^	Lag days	All seasons	Cold season	Warm season
			RERI (95% CI)	RERI (95% CI)	RERI (95% CI)

50th percentile	50th percentile	0–1	−0.08 (−0.24, 0.07)	0.11 (−0.13, 0.36)	−0.06 (−0.29, 0.16)
		0–3	0.06 (−0.09, 0.21)	0.10 (−0.16, 0.35)	0.18 (−0.02, 0.39)
		0–6	0.14 (−0.03, 0.30)	0.07 (−0.21, 0.35)	**0.26 (0.04, 0.49)**
75th percentile	75th percentile	0–1	0.01 (−0.20, 0.23)	0.43 (−0.10, 0.97)	0.06 (−0.19, 0.32)
		0–3	0.0004 (−0.23, 0.23)	0.04 (−0.43, 0.52)	0.21 (−0.05, 0.46)
		0–6	0.07 (−0.19, 0.32)	0.01 (−0.40, 0.42)	0.30 (−0.01, 0.61)
50th percentile	75th percentile	0–1	0.02 (−0.14, 0.18)	0.15 (−0.11, 0.42)	0.05 (−0.21, 0.31)
		0–3	0.03 (−0.14, 0.20)	0.08 (−0.21, 0.38)	**0.34 (0.11, 0.57)**
		0–6	0.04 (−0.16, 0.23)	−0.05 (−0.38, 0.27)	**0.37 (0.12, 0.63)**
75th percentile	50th percentile	0–1	−0.05 (−0.23, 0.13)	0.38 (−0.02, 0.77)	0.01 (−0.18, 0.21)
		0–3	0.04 (−0.15, 0.23)	0.28 (−0.11, 0.66)	0.10 (−0.11, 0.31)
		0–6	−0.05 (−0.26, 0.15)	0.06 (−0.24, 0.35)	0.001 (−0.25, 0.25)

Abbreviations: WBGT_max_, maximum wet bulb globe temperature; NO_2_, nitrogen dioxide; CI, confidence interval; RERI, relative excess risk due to interaction. Statistically significant (*P* < 0.05) associations are marked in bold.

a The 50th and 75th percentiles of the WBGT_max_ distribution for the total period, corresponding to 15 °C and 21 °C, respectively, were used to classify WBGT_max_ into a binary variable (>15 °C and ≤ 15 °C).

b The 50th and 75th percentiles of the NO_2_ distribution for the total period, corresponding to 30 ppb and 39 ppb, respectively, were used to classify NO_2_ into a binary variable.

## Data Availability

Data will be made available upon request following necessary compliance under current local institutional and state statutes and oversights.

## Appendix A. Supplementary data

Supplementary data to this article can be found online at https://doi.org/10.1016/j.envint.2026.110152.
