# Supplementary material for: Independent and interactive effects of wet bulb globe temperature and air pollution exposures on suicide mortality
Source: Environ Int. Author manuscript; Available in PMC 2026 Apr 6. (PMC13051550; doi:10.1016/j.envint.2026.110152)
Supplement: supplementary file [file NIHMS2153549-supplement-supplementary_file.docx]

Supplementary file

**Independent and interactive effects of wet bulb globe temperature and air pollution exposures on suicide mortality**

**Text S1** Calculation of wet bulb globe temperature incorporating other meteorological variables

**Table S1** Descriptive statistics for WBGT_max_ and air pollution for cases and control days, 2000–2016

**Fig. S1** Pearson’s correlation matrix plots between PM_2.5_, NO_2_, and WBGT_max_

**Table S2** Odds ratios of suicide mortality associated with each 5 ℃ increase in WBGT_max_ and 10 unit increase in PM_2.5_ or NO_2_ from single-and double-exposure models on a single-day lag

**Table S3** Multiplicative interactions of WBGT_max_ and air pollution for suicide mortality on a single-day lag

**Table S4** Relative excess risk due to interaction (RERI) of WBGT_max_ and PM_2.5_ on suicide mortality

**Table S5** Odds ratios and 95% confidence intervals for suicide mortality associated with a 5 °C increase in WBGT_max_ at different air pollutant levels on moving-average lag days

**Table S6** Odds ratios and 95% confidence intervals for suicide mortality associated with a 5 ℃ increase in WBGT_max_ at different air pollutant levels on a single-day lag

**Fig. S2** Exposure-response curves of associations between PM_2.5_, NO_2_, and WBGT_max_ (lag 0–3) and suicide mortality (lag 0–6)

**Fig. S3** Associations between air pollution and suicide with and without adjustment for co-pollutants

**Table S7** Associations of WBGT_max_ and air pollutants with suicide stratified by sex (lag 0-6)

**Table S8** Associations of WBGT_max_ and air pollutants with suicide stratified by age (lag 0-6)

**Table S9** Associations of WBGTmax and air pollutants with suicide stratified by region (lag 0-6)

**Table S10** Odds ratios of suicide mortality associated with each 5 °C increase in WBGT_max_ and 10 unit increase in PM_2.5_ and NO_2_ from single- and two-exposure models (lag 0-6)

**Table S11** Odds ratio and 95% confidence intervals for suicide mortality associated with a 5 ℃ increase in WBGT_max_ at different air pollution levels (lag 0-6)

**Table S12** Odds ratios of suicide mortality associated with each 10 unit increase in PM_2.5_ and NO_2_ on moving-average lag days during the warm season, excluding data from Independence Day (July 4th)

**Table S13** E-values (95% Confidence Interval) at odds ratio scale for the estimation of associations in main estimates (lag 0-6)

**Text S1** Calculation of wet bulb globe temperature incorporating other meteorological variables.

The wet bulb globe temperature (WBGT, in °C) takes into account air temperature, humidity, wind speed, and solar radiation to characterize the actual heat stress experienced by individuals outdoors. The WBGT is defined as a weighted average of natural wet bulb temperature ($T_{\omega}$, driven by humidity, wind speed, and solar radiation), black globe temperature ($T_{g}$, driven by solar radiation and wind speed), and air temperature ($T_{\alpha}$) (Liljegren et al., 2008). The formula used to calculate WBGT using the Liljegren model is:

$$WBGT=0.1T_{\alpha}+0.2T_{g}+0.7T_{\omega}$$

where $T_{\alpha}$ is the ambient temperature, $T_{g}$ is the black globe temperature, and $T_{\omega}$ is the natural wet bulb temperature. We selected the Liljegren approach because its algorithms are based on physical models of the heat energy balance of a globe and a wetted wick, and the approach has been found to be accurate (Ahn et al., 2024). The equation for $T_{g}$ is solved iteratively using ambient temperature, incoming solar radiation, solar zenith angle, and physical constants. The equation for $T_{g}$ is indicated as follows:

$$T_{g}= \sqrt[4]{\frac{1}{2}\left( 1+ \varepsilon_{\alpha} \right)T_{\alpha}^{4}- \frac{h}{\varepsilon_{g \sigma}} \left( T_{g}- T_{\alpha} \right) + \frac{S}{2\varepsilon_{g}\sigma} \left( 1- \alpha_{g} \right) \left[ 1 + \left( \frac{1}{2\cos(\theta)} - 1 \right) f_{dir} + \alpha_{sfc} \right]}$$

where $\varepsilon_{\alpha}:$ the emissivity of the air; $h$: the convective heat transfer coefficient; $\varepsilon_{g}$: the emissivity of the globe (set as 0.95); $S:$the total horizontal solar irradiance; $\alpha_{g}$: albedo of the ground (set as 0.05); $\theta$: the solar zenith angle (calculated from date, hour, longitude, and latitude); $f_{dir}$: the fraction of $S$ that is due to the direct beam of the sun; and $\alpha_{sfc}$: albedo of the surface.

The equation for $T_{\omega}$ is indicated as follows:

$$T_{w}=T_{\alpha}-\frac{\Delta H}{C_{p}} \frac{M_{H2O}}{M_{air}} \left( \frac{Pr}{Sc} \right)^{\alpha}\left( \frac{e_{\omega}- e_{\alpha}}{P- e_{\omega}} \right)+ \frac{{\Delta F}_{net}}{A h}$$

where $\Delta H:$the heat of vaporization; $C_{p}:$ the specific heat at constant pressure; $M_{H20}$: the molecular weight of water vapor; $M_{air}$: the molecular weight of the dry air; $Pr$: the Prandtl number; $S$c: the Schmidt number; $\alpha$: a constant = 0.56; $e_{\omega}$: the saturation vapor pressure of the wick; $e_{\alpha}$: the saturation vapor pressure of the air; $P$: the barometric pressure; ${\Delta F}_{net}$: the net radiant heat flux from the environment of the wick; $A$: the surface area of the wick; and $h$: the convective heat transfer coefficient.

**Table S1** Descriptive statistics for WBGT_max_ and air pollution for cases and control days, 2000–2016.

| Variables | Case days  (n = 7,551) | Control days (n = 25,657 |  | Mean difference (95% CI) |  | Percentiles (Total period) | | | | |
| --- | --- | --- | --- | --- | --- | --- | --- | --- | --- | --- |
|  | Mean (SD) | Mean (SD) |  |  |  | Min | P25 | P50 | P75 | Max |
| WBGT_max_ (°C) | |  |  |  |  |  |  |  |  |  |
| Lag 0 | 14.67 (8.00) | 14.60 (8.04) |  | –0.07 (–0.27, 0.14) |  | –13.59 | 8.26 | 15.33 | 21.59 | 31.45 |
| Lag 1 | 14.67 (8.02) | 14.63 (8.03) |  | –0.04 (–0.24, 0.17) |  | –10.97 | 8.26 | 15.35 | 21.61 | 31.85 |
| Lag 2 | 14.71 (7.96) | 14.63 (8.04) |  | –0.09 (–0.29, 0.12) |  | –11.02 | 8.27 | 15.30 | 21.61 | 31.87 |
| Lag 3 | 14.67 (7.97) | 14.63 (8.06) |  | –0.04 (–0.25, 0.16) |  | –12.55 | 8.25 | 15.40 | 21.65 | 31.85 |
| Lag 4 | 14.65 (8.03) | 14.65 (8.05) |  | 0.00 (–0.21, 0.21) |  | –13.70 | 8.23 | 15.38 | 21.65 | 31.91 |
| Lag 5 | 14.61 (8.05) | 14.65 (8.04) |  | 0.05 (–0.16, 0.25) |  | –12.10 | 8.23 | 15.41 | 21.64 | 31.53 |
| Lag 6 | 14.63 (8.0) | 14.65 (8.02) |  | 0.03 (–0.18, 0.23) |  | –13.09 | 8.24 | 15.39 | 21.64 | 31.90 |
| Lag 0–6 | 14.65 (7.63) | 14.63 (7.67) |  | –0.02 (–0.22, 0.17) |  | –6.19 | 8.30 | 15.14 | 21.42 | 30.24 |
|  |  |  |  |  |  |  |  |  |  |  |
| PM_2.5_ (μg/m^3^) | |  |  |  |  |  |  |  |  |  |
| Lag 0 | 8.21 (8.75) | 8.26 (8.67) |  | 0.05 (–0.17, 0.27) |  | 0.23 | 4.06 | 5.86 | 8.70 | 171.20 |
| Lag 1 | 8.24 (8.77) | 8.26 (8.88) |  | 0.02 (–0.21, 0.25) |  | 0.17 | 4.08 | 5.88 | 8.67 | 180.26 |
| Lag 2 | 8.34 (9.01) | 8.24 (8.77) |  | –0.09 (–0.32, 0.13) |  | 0.15 | 4.09 | 5.92 | 8.73 | 189.31 |
| Lag 3 | 8.39 (9.06) | 8.25 (8.87) |  | –0.13 (–0.36, 0.10) |  | 0.16 | 4.10 | 5.91 | 8.71 | 188.64 |
| Lag 4 | 8.36 (9.17) | 8.26 (8.90) |  | –0.10 (–0.33, 0.13) |  | 0.17 | 4.11 | 5.93 | 8.73 | 196.47 |
| Lag 5 | 8.34 (8.97) | 8.31 (9.01) |  | –0.03 (–0.26, 0.20) |  | 0.20 | 4.10 | 5.90 | 8.74 | 193.92 |
| Lag 6 | 8.37 (9.04) | 8.30 (8.96) |  | –0.07 (–0.30, 0.16) |  | 0.19 | 4.09 | 5.90 | 8.67 | 193.51 |
| Lag 0–6 | 8.33 (7.63) | 8.28 (7.54) |  | –0.05 (–0.24, 0.14) |  | 0.22 | 4.59 | 6.22 | 8.86 | 163.35 |
|  |  |  |  |  |  |  |  |  |  |  |
| NO_2_ (ppb) |  |  |  |  |  |  |  |  |  |  |
| Lag 0 | 30.06 (14.39) | 30.03 (14.46) |  | –0.03 (–0.40, 0.34) |  | 0.38 | 18.98 | 29.72 | 39.98 | 162.31 |
| Lag 1 | 30.05 (14.30) | 29.87 (14.39) |  | –0.18 (–0.55, 0.19) |  | 0.40 | 18.86 | 29.66 | 39.83 | 135.23 |
| Lag 2 | 30.11 (14.40) | 29.89 (14.33) |  | –0.22 (–0.59, 0.15) |  | 0.40 | 18.89 | 29.79 | 39.97 | 133.95 |
| Lag 3 | 30.17 (14.42) | 30.01 (14.27) |  | –0.16 (–0.53, 0.21) |  | 0.50 | 19.03 | 30.03 | 39.94 | 138.30 |
| Lag 4 | 30.18 (14.39) | 30.03 (14.28) |  | –0.15 (–0.52, 0.21) |  | 0.50 | 18.93 | 30.00 | 40.02 | 154.22 |
| Lag 5 | 30.17 (14.43) | 30.11 (14.35) |  | –0.05 (–0.42, 0.32) |  | 0.79 | 19.00 | 30.05 | 40.18 | 154.00 |
| Lag 6 | 30.20 (14.42) | 30.10 (14.40) |  | –0.11 (–0.48, 0.26) |  | 0.63 | 19.12 | 29.95 | 40.14 | 175.45 |
| Lag 0–6 | 30.14 (12.90) | 30.01 (12.88) |  | –0.13 (–0.46, 0.20) |  | 0.75 | 20.70 | 29.99 | 39.12 | 145.58 |

Abbreviations: SD, standard deviation; WBGT_max_, maximum wet bulb globe temperature; PM_2.5_, particulate matter with aerodynamic diameter ≤2.5 µm; NO_2_, nitrogen dioxide; P, percentile; ppb, parts per billion.


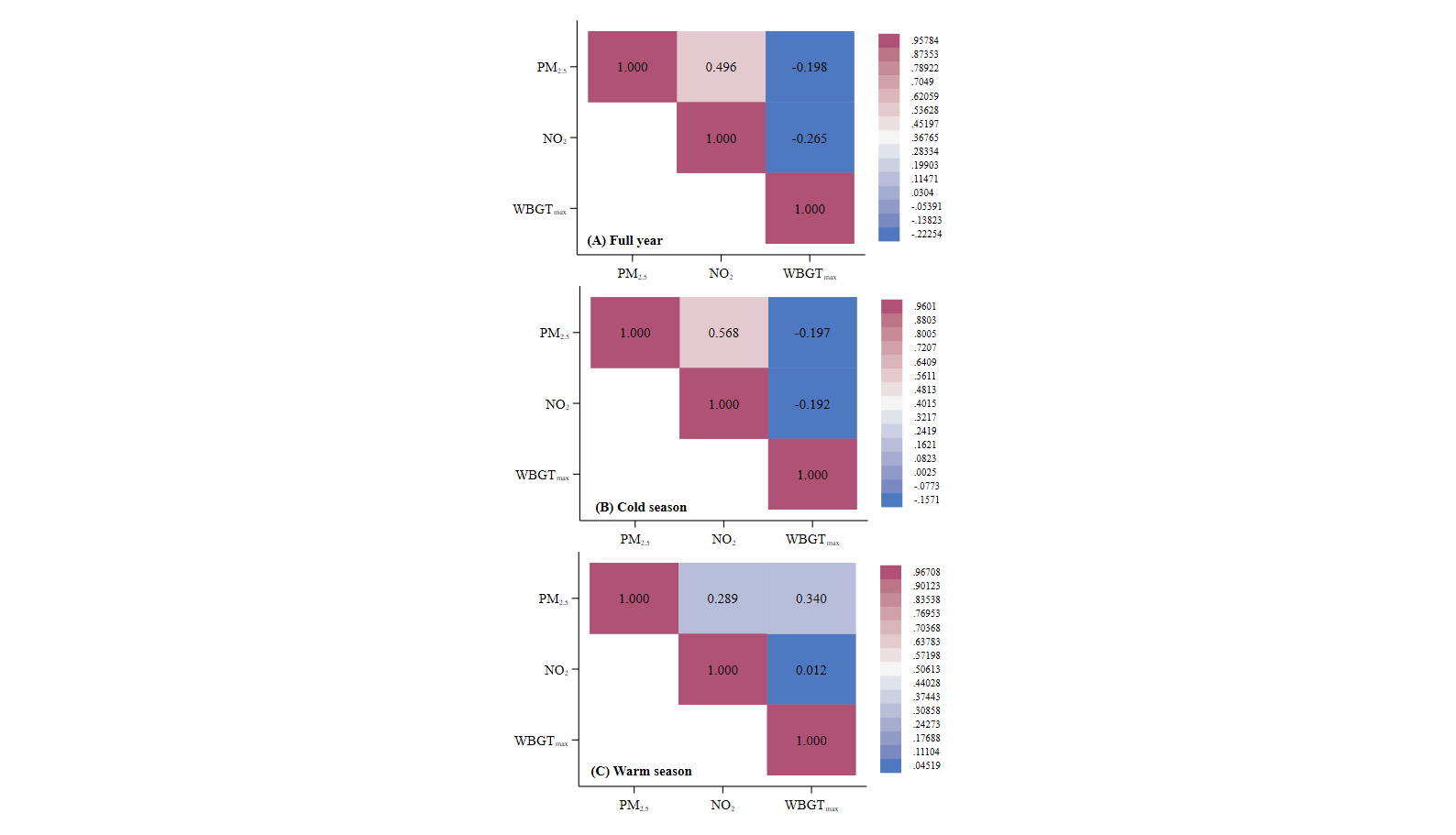


**Fig. S1.** Pearson’s correlation matrix plots between PM_2.5_, NO_2_, and WBGT_max_. Abbreviations: WBGT_max_, maximum wet bulb globe temperature; PM_2.5_, particulate matter with aerodynamic diameter ≤2.5 µm; NO_2_, nitrogen dioxide.

**Table S2** Odds ratios of suicide mortality associated with each 5 °C increase in WBGT_max_ and 10 unit increase in PM_2.5_ or NO_2_ from single-and two-exposure models on a single-day lag.

| Models | Exposure | Season | Lag 0 | Lag 1 | Lag 2 | Lag 3 | Lag 4 | Lag 5 | Lag 6 |
| --- | --- | --- | --- | --- | --- | --- | --- | --- | --- |
|  |  |  | OR (95% CI) | OR (95% CI) | OR (95% CI) | OR (95% CI) | OR (95% CI) | OR (95% CI) | OR (95% CI) |
| Single-exposure model^a^ | PM_2.5_ | All seasons | 0.99 (0.95, 1.03) | 1.00 (0.96, 1.03) | 1.02 (0.98, 1.06) | 1.03 (0.99, 1.06) | 1.02 (0.99, 1.06) | 1.01 (0.97, 1.04) | 1.01 (0.98, 1.05) |
|  |  | Cold season | 1.01 (0.97, 1.05) | 1.01 (0.97, 1.05) | 1.03 (0.99, 1.07) | **1.03 (1.00, 1.07)** | 1.03 (0.99, 1.07) | 1.01 (0.97, 1.05) | 1.01 (0.97, 1.05) |
|  |  | Warm season | **0.83 (0.74, 0.95)** | 0.89 (0.79, 1.00) | 0.92 (0.81, 1.03) | 0.97 (0.87, 1.09) | 0.94 (0.84, 1.06) | 0.98 (0.87, 1.09) | 1.03 (0.93, 1.15) |
|  | NO_2_ | All seasons | 1.00 (0.97, 1.03) | 1.03 (0.99, 1.06) | **1.03 (1.00, 1.07)** | 1.02 (0.99, 1.06) | 1.02 (0.99, 1.06) | 1.01 (0.97, 1.04) | 1.01 (0.98, 1.05) |
|  |  | Cold season | 1.03 (0.99, 1.08) | **1.06 (1.01, 1.11)** | **1.07 (1.02, 1.12)** | **1.07 (1.02, 1.12)** | **1.07 (1.02, 1.11)** | **1.04 (1.00, 1.09)** | **1.04 (1.00, 1.09)** |
|  |  | Warm season | 0.95 (0.90, 1.00) | 0.98 (0.93, 1.03) | 0.99 (0.94, 1.04) | 0.96 (0.91, 1.01) | 0.96 (0.91, 1.01) | 0.96 (0.91, 1.01) | 0.97 (0.93, 1.03) |
|  | WBGT_max_ | All seasons | **1.04 (1.00, 1.08)** | 1.03 (0.99, 1.07) | **1.05 (1.01, 1.09)** | 1.03 (0.99, 1.07) | 1.01 (0.97, 1.05) | 0.99 (0.96, 1.03) | 1.00 (0.96, 1.03) |
|  |  | Cold season | 1.01 (0.96, 1.06) | 0.98 (0.94, 1.03) | 1.02 (0.98, 1.07) | 1.01 (0.96, 1.06) | 0.97 (0.92, 1.01) | **0.93 (0.88, 0.97)** | **0.93 (0.89, 0.98)** |
|  |  | Warm season | **1.08 (1.02, 1.15)** | **1.10 (1.04, 1.17)** | **1.09 (1.03, 1.16)** | **1.06 (1.00, 1.13)** | **1.08 (1.02, 1.15)** | **1.10 (1.04, 1.16)** | **1.10 (1.04, 1.16)** |
| Two-exposure model  (PM_2.5_ and WBGT_max_)^b^ |  |  |  |  |  |  |  |  |  |
|  | PM_2.5_ | All seasons | 0.98 (0.95, 1.02) | 0.99 (0.95, 1.03) | 1.01 (0.97, 1.05) | 1.02 (0.98, 1.06) | 1.02 (0.98, 1.06) | 1.01 (0.97, 1.05) | 1.01 (0.98, 1.05) |
|  | WBGT_max_ | All seasons | **1.04 (1.00, 1.08)** | 1.03 (0.99, 1.07) | **1.05 (1.01, 1.09)** | 1.03 (0.99, 1.07) | 1.01 (0.97, 1.04) | 0.99 (0.95, 1.03) | 0.99 (0.96, 1.03) |
|  | PM_2.5_ | Cold season | 1.01 (0.97, 1.05) | 1.01 (0.97, 1.05) | 1.03 (0.99, 1.07) | 1.03 (0.99, 1.07) | **1.04 (1.00, 1.08)** | 1.02 (0.98, 1.06) | 1.02 (0.98, 1.06) |
|  | WBGT_max_ | Cold season | 1.01 (0.96, 1.06) | 0.98 (0.93, 1.03) | 1.02 (0.97, 1.07) | 1.00 (0.96, 1.05) | 0.96 (0.91, 1.00) | **0.92 (0.88, 0.97)** | **0.93 (0.89, 0.98)** |
|  | PM_2.5_ | Warm season | **0.79 (0.69, 0.90)** ^†^ | **0.83 (0.74, 0.95)** ^†^ | **0.86 (0.76, 0.98)** ^†^ | 0.94 (0.83, 1.06) | 0.90 (0.79, 1.01) ^†^ | 0.93 (0.82, 1.05) | 0.99 (0.89, 1.11) |
|  | WBGT_max_ | Warm season | **1.12 (1.05, 1.19)** ^†^ | **1.13 (1.06, 1.20)**^†^ | **1.11 (1.05, 1.18)** ^†^ | **1.07 (1.01, 1.14)** | **1.10 (1.03, 1.17)**^†^ | **1.11 (1.04, 1.18)** ^†^ | **1.10 (1.03, 1.17)** ^†^ |
|  |  |  |  |  |  |  |  |  |  |
| Two-exposure model  (NO_2_ and WBGT_max_)^c^ | NO_2_ | All seasons | 0.99 (0.96, 1.03) | 1.02 (0.99, 1.06) | 1.03 (0.99, 1.06) | 1.02 (0.98, 1.05) | 1.02 (0.99, 1.06) | 1.01 (0.98, 1.04) | 1.02 (0.98, 1.05) |
|  | WBGT_max_ | All seasons | **1.04 (1.00, 1.08)** | 1.02 (0.98, 1.06) | **1.05 (1.01, 1.09)** | 1.03 (0.99, 1.07) | 1.01 (0.97, 1.04) | 0.99 (0.95, 1.03) | 0.99 (0.96, 1.03) |
|  | NO_2_ | Cold season | 1.03 (0.99, 1.08) | **1.07 (1.02, 1.11)** | **1.07 (1.02, 1.11)** | **1.07 (1.02, 1.12)** | **1.08 (1.03, 1.13)** | **1.06 (1.01, 1.11)** | **1.06 (1.01, 1.11)** |
|  | WBGT_max_ | Cold season | 1.00 (0.96, 1.05) | 0.97 (0.92, 1.02) | 1.01 (0.96, 1.06) | 0.99 (0.95, 1.04) | **0.95 (0.90, 0.99)** | 0.91 (0.87, 0.96) | **0.92 (0.88, 0.97)** |
|  | NO_2_ | Warm season | **0.93 (0.88, 0.98)**^†^ | 0.95 (0.90, 1.01) ^†^ | 0.97 (0.92, 1.02) ^†^ | 0.95 (0.90, 1.00) ^†^ | **0.94 (0.89, 0.99)** ^†^ | **0.94 (0.89, 0.99**)^†^ | 0.95 (0.90, 1.00) ^†^ |
|  | WBGT_max_ | Warm season | **1.11 (1.04, 1.18)**^†^ | **1.12 (1.05, 1.19**) ^†^ | **1.10 (1.04, 1.17)** ^†^ | **1.08 (1.02, 1.15)** | **1.10 (1.04, 1.17)**^†^ | **1.12 (1.05, 1.19)** ^†^ | **1.11 (1.05, 1.18)**^†^ |

All models adjusted for public holidays. Abbreviations: WBGT_max_, maximum wet bulb globe temperature; PM_2.5_, particulate matter with aerodynamic diameter ≤2.5 µm; NO_2_, nitrogen dioxide; OR, odds ratio; CI, confidence interval.

^a^ This model included only WBGT_max_, PM_2.5_, or NO_2_ to test the main effect of each 5 °C increase in WBGT_max_ or 10 unit increase in PM_2.5_ or NO_2_ on suicide mortality.

^b^ This model included WBGT_max_ and PM_2.5_ simultaneously to test the main effects of each 5 °C increase in WBGT_max_ and each 10 µg/m^3^ increase in PM_2.5_ on suicide mortality.

^c^ This model included WBGT_max_ and NO_2_ simultaneously to test the main effects of each 5 °C increase in WBGT_max_ and each 10 ppb increase in NO_2_ on suicide mortality.

^†^ Differences between the two odds ratios (ORs) of subgroup analysis (cold and warm seasons) in the double-exposure models are statistically significant.

Statistically significant (*P* <0.05) associations are marked in bold.

**Table S3** Multiplicative interactions of WBGT_max_ and air pollution for suicide mortality on a single-day lag.

| Exposure | Lag days | All seasons |  | Cold season |  | Warm season |
| --- | --- | --- | --- | --- | --- | --- |
|  |  | OR (95% CI)^c^ |  | OR (95% CI)^c^ |  | OR (95% CI)^c^ |
| PM_2.5_ and WBGT_max_^a^ | 0 | 0.98 (0.95, 1.00) |  | 1.02 (0.98, 1.06) |  | 1.01 (0.91, 1.13) |
|  | 1 | 0.98 (0.95, 1.01) |  | 1.01 (0.97, 1.05) |  | 1.01 (0.91, 1.12) |
|  | 2 | 0.99 (0.96, 1.02) |  | 1.02 (0.98, 1.07) |  | 1.07 (0.96, 1.19) |
|  | 3 | 0.99 (0.97, 1.02) |  | 0.99 (0.95, 1.04) |  | **1.14 (1.03, 1.26)** |
|  | 4 | 0.99 (0.96, 1.02) |  | 1.01 (0.97, 1.05) |  | 1.09 (0.98, 1.21) |
|  | 5 | 1.00 (0.97, 1.03) |  | 1.03 (0.99, 1.08) |  | 1.01 (0.92, 1.11) |
|  | 6 | 1.00 (0.97, 1.03) |  | 1.01 (0.97, 1.06) |  | 1.03 (0.94, 1.13) |
|  |  |  |  |  |  |  |
| NO_2_ and WBGT_max_^b^ | 0 | 1.00 (0.99, 1.02) |  | **1.04 (1.01, 1.06)** |  | 1.03 (0.99, 1.07) |
|  | 1 | 1.01 (0.99, 1.02) |  | **1.04 (1.01, 1.08)** |  | **1.04 (1.01, 1.08)** |
|  | 2 | 1.01 (0.99, 1.03) |  | 1.03 (0.99, 1.05) |  | **1.06 (1.02, 1.10)** |
|  | 3 | 1.01 (0.99, 1.02) |  | **1.03 (1.00, 1.06)** |  | **1.06 (1.02, 1.10)** |
|  | 4 | 0.99 (0.98, 1.02) |  | 1.02 (0.99, 1.04) |  | **1.05 (1.02, 1.09)** |
|  | 5 | 1.01 (0.99, 1.03) |  | 1.02 (0.99, 1.05) |  | **1.08 (1.04, 1.11)** |
|  | 6 | 1.00 (0.99, 1.02) |  | 1.01 (0.98, 1.04) |  | **1.07 (1.04, 1.11)** |

All models were adjusted for public holidays. Abbreviations: WBGT_max_, maximum wet bulb globe temperature; PM_2.5_, particulate matter with aerodynamic diameter ≤2.5 µm; NO_2_, nitrogen dioxide; OR, odds ratio; CI, confidence interval.

^a^ Interaction term (WBGT_max_ × PM_2.5_) was added into the two-exposure model to test the interaction between each 5 °C increase in WBGT_max_ and each 10 µg/m^3^ increase in PM_2.5_ on suicide mortality.

^b^ Interaction term (WBGT_max_ × NO_2_) was added into the two-exposure model to test the interaction between each 5 °C increase in WBGT_max_ and each 10 ppb increase in NO_2_ on suicide mortality.

^c^ OR and 95% CI for the interaction term. A significant interaction term indicates the existence of multiplicative interaction.

Statistically significant (*P* <0.05) associations are marked in bold.

**Table S4** Relative excess risk due to interaction (RERI) of WBGT_max_ and PM_2.5_ on suicide mortality.

| WBGT_max_^a^ | PM_2.5_^b^ | Lag days | All seasons |  | Cold season |  | Warm season |
| --- | --- | --- | --- | --- | --- | --- | --- |
|  |  |  | RERI (95% CI) |  | RERI (95% CI) |  | RERI (95% CI) |
|  |  |  |  |  |  |  |  |
| 50^th^ percentile | 50^th^ percentile | 0-1 | –0.01 (–0.14, 0.12) |  | **0.23 (0.01, 0.45)** |  | 0.03 (–0.19, 0.24) |
|  |  | 0-3 | –0.07 (–0.20, 0.06) |  | –0.04 (–0.27, 0.19) |  | 0.002 (–0.23, 0.23) |
|  |  | 0-6 | –0.02 (–0.16, 0.12) |  | –0.03 (–0.27, 0.21) |  | 0.002 (–0.24, 0.24) |
|  |  |  |  |  |  |  |  |
| 75^th^ percentile | 75^th^ percentile | 0-1 | –0.01 (–0.18, 0.15) |  | –0.08 (–0.52, 0.36) |  | 0.20 (–0.02, 0.41) |
|  |  | 0-3 | –0.14 (–0.31, 0.03) |  | 0.10 (–0.49, 0.68) |  | 0.09 (–0.15, 0.32) |
|  |  | 0-6 | –0.15 (–0.32, 0.03) |  | –0.23 (–0.58, 0.11) |  | 0.22 (–0.01, 0.46) |
|  |  |  |  |  |  |  |  |
| 50^th^ percentile | 75^th^ percentile | 0-1 | –0.004 (–0.15, 0.14) |  | 0.19 (–0.10, 0.48) |  | 0.22 (–0.07, 0.50) |
|  |  | 0-3 | –0.09 (–0.25, 0.06) |  | 0.09 (–0.23, 0.42) |  | **0.29 (0.01, 0.57)** |
|  |  | 0-6 | **–0.21 (–0.38, –0.04)** |  | –0.26 (–0.59, 0.08) |  | **0.30 (0.02, 0.57)** |
|  |  |  |  |  |  |  |  |
| 75^th^ percentile | 50^th^ percentile | 0-1 | –0.01 (–0.16, 0.14) |  | 0.17 (–0.18, 0.52) |  | 0.08 (–0.10, 0.25) |
|  |  | 0-3 | 0.02 (–0.13, 0.17) |  | –0.06 (–0.40, 0.28) |  | 0.12 (–0.06, 0.29) |
|  |  | 0-6 | 0.02 (–0.13, 0.18) |  | 0.06 (–0.19, 0.30) |  | 0.07 (–0.12, 0.26) |

Abbreviations: WBGT_max_, maximum wet bulb globe temperature; PM_2.5_, particulate matter with aerodynamic diameter ≤2.5 µm; CI, confidence interval; RERI, relative excess risk due to interaction.

^a^ The 50^th^ and 75^th^ percentiles of the WBGT_max_ distribution for the total period, corresponding to 15 °C and 21 °C, respectively, were used to classify WBGT_max_ into a binary variable.

^b^ The 50^th^ and 75th percentiles of the PM_2.5_ distribution for the total period, corresponding to 6 µg/m^3^ and 9 µg/m^3^, respectively, were used to classify PM_2.5_ into a binary variable.

Statistically significant (*P* <0.05) associations are marked in bold.

**Table S5** Odds ratios and 95% confidence intervals for suicide mortality associated with a 5 °C increase in WBGT_max_ at different air pollutant levels on moving-average lag days.

| Season/modifier | Air pollution levels | Lag 0-1 | Lag 0-3 | Lag 0-6 |
| --- | --- | --- | --- | --- |
|  |  | OR (95% CI) | OR (95% CI) | OR (95% CI) |
| *All seasons* |  |  |  |  |
| PM_2.5_ | Low | 0.99 (0.92, 1.08) | 1.03 (0.94, 1.13) | 1.09 (0.98, 1.21) |
|  | Moderate | 1.05 (0.96, 1.16) | 1.06 (0.95, 1.18) | 1.08 (0.96, 1.23) |
|  | High | 1.04 (0.96, 1.14) | 1.06 (0.97, 1.16) | 0.98 (0.88, 1.08) |
|  | Difference test | 0.05 (–0.07, 0.16) | 0.03 (–0.10, 0.16) | –0.11 (–0.26, 0.04) |
|  |  |  |  |  |
| NO_2_ | Low | 0.99 (0.92, 1.08) | 1.00 (0.91, 1.09) | 0.97 (0.87, 1.08) |
|  | Moderate | 1.07 (0.99, 1.16) | 1.11 (1.01, 1.21) | 1.10 (1.00, 1.21) |
|  | High | 1.02 (0.94, 1.10) | 1.07 (0.99, 1.16) | 1.02 (0.93, 1.11) |
|  | Difference test | 0.02 (–0.09, 0.13) | 0.07 (–0.05. 0.20) | 0.05 (–0.09, 1.19) |
| *Cold season* |  |  |  |  |
| PM_2.5_ | Low | 0.93 (0.84, 1.03) | 0.99 (0.88, 1.11) | 0.96 (0.84, 1.10) |
|  | Moderate | 0.99 (0.86, 1.14) | 1.01 (0.87, 1.18) | 0.94 (0.79, 1.12) |
|  | High | 1.02 (0.93, 1.12) | 1.03 (0.94, 1.14) | 0.95 (0.86, 1.06) |
|  | Difference test | 0.09 (–0.04, 0.23) | 0.05 (–0.11, 0.20) | –0.00 (–0.18, 0.17) |
|  |  |  |  |  |
| NO_2_ | Low | 0.95 (0.85, 1.06) | 0.95 (0.83, 1.08) | **0.84 (0.72, 0.97)** |
|  | Moderate | 1.02 (0.92, 1.14) | 1.03 (0.92, 1.16) | 1.03 (0.91, 1.17) |
|  | High | 0.98 (0.90, 1.06) | 1.01 (0.92, 1.10) | 0.94 (0.85, 1.04) |
|  | Difference test | 0.03 (–0.11, 0.17) | 0.06 (–0.10, 0.22) | 0.12 (–0.06, 0.30) |
| *Warm season* |  |  |  |  |
| PM_2.5_ | Low | 1.11 (0.98, 1.27) | 1.10 (0.95, 1.27) | **1.30 (1.11, 1.52)** |
|  | Moderate | 1.12 (0.97, 1.28) | 1.11 (0.95, 1.30) | **1.25 (1.05, 1.49)** |
|  | High | 1.22 (0.96, 1.56) | 1.29 (0.98, 1.70) | 1.21 (0.88, 1.67) |
|  | Difference test | 0.09 (–0.18, 0.37) | 0.16 (–0.15, 0.47) | –0.07 (–0.42, 0.29) |
|  |  |  |  |  |
| NO_2_ | Low | 1.04 (0.93, 1.16) | 1.04 (0.92, 1.18) | 1.11 (0.96, 1.28) |
|  | Moderate | **1.15 (1.01, 1.31)** | **1.23 (1.07, 1.41)** | **1.22 (1.05, 1.42)** |
|  | High | 1.17 (0.99, 1.39) | **1.40 (1.16, 1.69)** | **1.48 (1.19, 1.84)** |
|  | Difference test | 0.12 (–0.08, 0.32) | 0.29 (0.07, 0.52)^†^ | 0.29 (0.03, 0.55)^†^ |

Abbreviations: WBGT_max_, maximum wet bulb globe temperature; PM_2.5_, particulate matter with aerodynamic diameter ≤2.5 µm; NO_2_, nitrogen dioxide; OR, odds ratio; CI, confidence interval.

^†^ Differences between the two coefficients of subgroup (high and low PM_2.5_ or NO_2_ levels) are statistically significant at *P* < 0.05.

Statistically significant (*P* <0.05) associations are marked in bold.

**Table S6** Odds ratios and 95% confidence intervals for suicide mortality associated with a 5 °C increase in WBGT_max_ at different air pollutant levels on a single-day lag.

| Season/Modifier | Air pollution levels | Lag 0 | Lag 1 | Lag 2 | Lag 3 | Lag 4 | Lag 5 | Lag 6 |
| --- | --- | --- | --- | --- | --- | --- | --- | --- |
|  |  | OR (95% CI) | OR (95% CI) | OR (95% CI) | OR (95% CI) | OR (95% CI) | OR (95% CI) | OR (95% CI) |
| *All seasons* |  |  |  |  |  |  |  |  |
| PM_2.5_ | Low | 1.00 (0.93, 1.08) | 0.99 (0.92, 1.06) | 1.02 (0.95, 1.10) | 1.06 (0.99, 1.15) | 1.05 (0.98, 1.13) | 0.99 (0.92, 1.06) | 1.02 (0.95, 1.09) |
|  | Moderate | **1.10 (1.00, 1.20)** | 1.03 (0.93, 1.13) | 1.01 (0.92, 1.11) | 1.05 (0.95, 1.15) | 0.91 (0.83, 1.00) | 1.02 (0.93, 1.12) | 0.99 (0.91, 1.09) |
|  | High | 1.04 (0.96, 1.13) | 1.00 (0.93, 1.09) | 1.04 (0.96, 1.13) | 1.00 (0.92, 1.08) | 0.94 (0.87, 1.02) | 0.95 (0.88, 1.03) | 0.99 (0.91, 1.07) |
|  | Difference test | 0.04 (–0.07, 0.15) | 0.02 (–0.09, 0.13) | 0.01 (–0.10, 0.12) | –0.06 (–0.17, 0.05) | 0.11 (–0.22, 0.00) | –0.04 (–0.14, 0.07) | –0.03 (–0.14, 0.08) |
|  |  |  |  |  |  |  |  |  |
| NO_2_ | Low | 0.99 (0.92, 1.07) | 1.02 (0.95, 1.11) | 1.04 (0.96, 1.12) | 1.00 (0.92, 1.08) | 0.99 (0.92, 1.07) | **0.92 (0.85, 0.99)** | 0.95 (0.88, 1.03) |
|  | Moderate | 1.07 (0.99, 1.16) | 1.04 (0.96, 1.12) | **1.12 (1.04, 1.21)** | 1.05 (0.97, 1.13) | 1.06 (0.99, 1.15) | 1.06 (0.99, 1.15) | 1.05 (0.97, 1.14) |
|  | High | 1.04 (0.97, 1.12) | 1.00 (0.93, 1.08) | 1.05 (0.98, 1.13) | 0.99 (0.92, 1.06) | 0.98 (0.92, 1.05) | 1.00 (0.93, 1.07) | 1.00 (0.93, 1.07) |
|  | Difference test | 0.05 (–0.06, 0.15) | –0.02 (–0.13, 0.08) | 0.01 (–0.09, 0.11) | –0.01 (–0.11, 0.09) | –0.01 (–0.11, 0.09) | 0.08 (–0.02, 0.18) | 0.05 (–0.05, 0.15) |
| *Cold season* |  |  |  |  |  |  |  |  |
| PM_2.5_ | Low | 0.93 (0.85, 1.03) | **0.91 (0.83, 0.99)** | 1.04 (0.94, 1.14) | 1.04 (0.95, 1.15) | 1.04 (0.94, 1.15) | **0.88 (0.79, 0.97)** | 0.92 (0.83, 1.01) |
|  | Moderate | 1.07 (0.94, 1.21) | 0.98 (0.85, 1.12) | 0.95 (0.84, 1.09) | 1.08 (0.94, 1.25) | **0.79 (0.69, 0.91)** | 0.99 (0.87, 1.13) | **0.86 (0.76, 0.99)** |
|  | High | 1.03 (0.94, 1.12) | 0.99 (0.90, 1.08) | 0.99 (0.91, 1.08) | 0.97 (0.89, 1.05) | 0.93 (0.86, 1.02) | 0.95 (0.87, 1.03) | 0.96 (0.87, 1.05) |
|  | Difference test | 0.10 (–0.03, 0.23) | 0.08 (–0.05, 0.22) | –0.04 (–0.18, 0.09) | –0.08 (–0.21, 0.05) | –0.11 (–0.24, 0.02) | 0.08 (–0.05, 0.21) | 0.04 (–0.10, 0.17) |
|  |  |  |  |  |  |  |  |  |
| NO_2_ | Low | 0.94 (0.84, 1.05) | 0.98 (0.88, 1.10) | 0.98 (0.87, 1.10) | 0.95 (0.84, 1.06) | 0.89 (0.79, 1.00) | **0.84 (0.75, 0.94)** | **0.88 (0.79, 0.98)** |
|  | Moderate | 1.03 (0.92, 1.14) | 0.98 (0.89, 1.08) | **1.12 (1.01, 1.24)** | 1.04 (0.94, 1.16) | 1.04 (0.94, 1.16) | 1.00 (0.90, 1.10) | 1.04 (0.93, 1.15) |
|  | High | 1.01 (0.94, 1.10) | 0.96 (0.88, 1.04) | 1.00 (0.92, 1.08) | 0.96 (0.88, 1.03) | 0.94 (0.87, 1.01) | 0.94 (0.87, 1.02) | 0.93 (0.86, 1.00) |
|  | Difference test | 0.08 (–0.06, 0.21) | –0.02 (–0.16, 0.11) | 0.02 (–0.13, 0.16) | 0.01 (–0.13, 0.15) | 0.05 (–0.08, 0.19) | 0.11 (–0.02, 0.25) | 0.05 (–0.08, 0.19) |
| *Warm season* |  |  |  |  |  |  |  |  |
| PM_2.5_ | Low | 1.12 (0.99, 1.26) | 1.12 (0.99, 1.26) | 1.01 (0.89, 1.13) | 1.09 (0.97, 1.22) | 1.06 (0.95, 1.18) | **1.15 (1.03, 1.28)** | **1.16 (1.04, 1.29)** |
|  | Moderate | 1.13 (0.99, 1.29) | 1.07 (0.94, 1.22) | 1.08 (0.94, 1.23) | 1.02 (0.89, 1.16) | 1.02 (0.90, 1.17) | 1.06 (0.92, 1.21) | 1.13 (0.99, 1.29) |
|  | High | 1.11 (0.90, 1.38) | 1.12 (0.91, 1.39) | **1.34 (1.09, 1.64)** | 1.21 (0.99, 1.48) | 1.03 (0.84, 1.27) | 0.98 (0.81, 1.18) | 1.12 (0.93, 1.36) |
|  | Difference test | –0.004 (–0.25, 0.24) | 0.001 (–0.24, 0.24) | 0.28 (0.05, 0.52)^†^ | 0.11 (–0.13, 0.34) | –0.03 (–0.27, 0.21) | –0.16 (–0.39, 0.06) | –0.03 (–0.26, 0.19) |
|  |  |  |  |  |  |  |  |  |
| NO_2_ | Low | 1.04 (0.94, 1.16) | 1.06 (0.96, 1.18) | 1.08 (0.98, 1.20) | 1.04 (0.94, 1.15) | 1.08 (0.97, 1.19) | 0.98 (0.89, 1.09) | 1.01 (0.91, 1.12) |
|  | Moderate | **1.14 (1.01, 1.30)** | **1.14 (1.01, 1.28)** | 1.12 (0.99, 1.25) | 1.05 (0.94, 1.19) | 1.09 (0.97, 1.23) | **1.16 (1.03, 1.31)** | 1.08 (0.96, 1.22) |
|  | High | 1.15 (0.98, 1.35) | **1.17 (1.01, 1.37)** | **1.31 (1.12, 1.55)** | 1.11 (0.95, 1.30) | **1.17 (1.01, 1.36)** | **1.24 (1.07, 1.44)** | **1.31 (1.13, 1.52)** |
|  | Difference test | 0.10 (–0.09, 0.29) | 0.10 (–0.09, 0.28) | 0.19 (–0.001, 0.38) | 0.07 (–0.11, 0.26) | 0.08 (–0.10, 0.26) | 0.23 (0.05, 0.41)^†^ | 0.26 (0.08, 0.44)^†^ |

All models adjusted for public holidays. Abbreviations: WBGT_max_, maximum wet bulb globe temperature; PM_2.5_, particulate matter with aerodynamic diameter ≤2.5 µm; NO_2_, nitrogen dioxide; OR, odds ratio; CI, confidence interval.

^†^ Differences between the two coefficients of subgroup (high and low PM_2.5_ or NO_2_) are statistically significant at *P* < 0.05.

Statistically significant (*P* <0.05) associations are marked in bold.


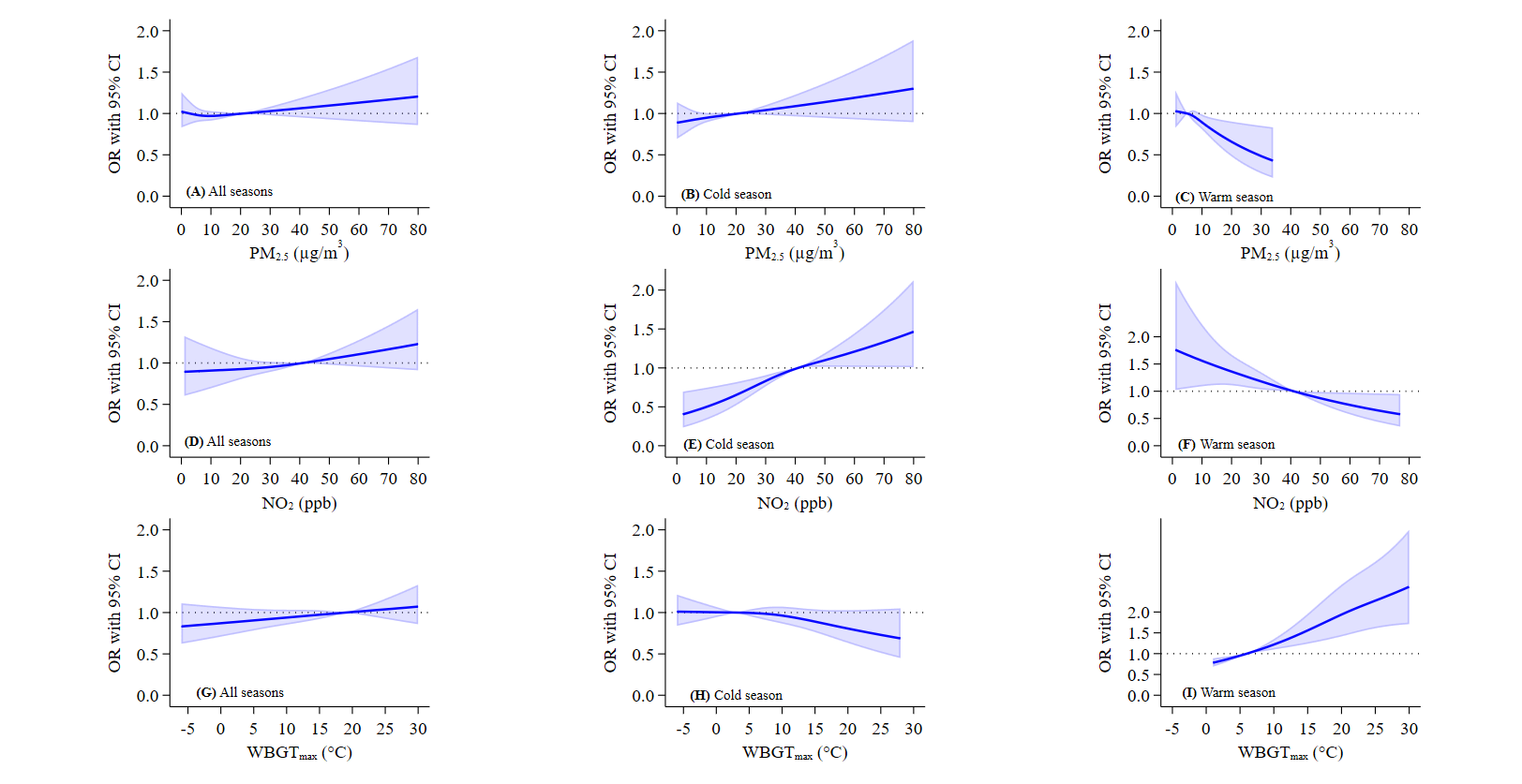


**Fig. S2.** Exposure-response curves of associations between PM_2.5_, NO_2_, and WBGT_max_ and suicide mortality (lag 0–6). The solid blue lines with shaded areas represent the odds ratios and their 95% confidence intervals (CI). This curve was calculated using the restricted cubic splines with knots at the 10^th^, 50^th^, and 90^th^ percentiles. Figures A–C for PM_2.5,_ D–F for NO_2,_ and G–I for WBGT_max_. Abbreviations: WBGT_max_, maximum wet bulb globe temperature; PM_2.5_, particulate matter with aerodynamic diameter ≤2.5 µm; NO_2_, nitrogen dioxide; ppb, parts per billion; OR, odds ratio; CI, confidence interval.


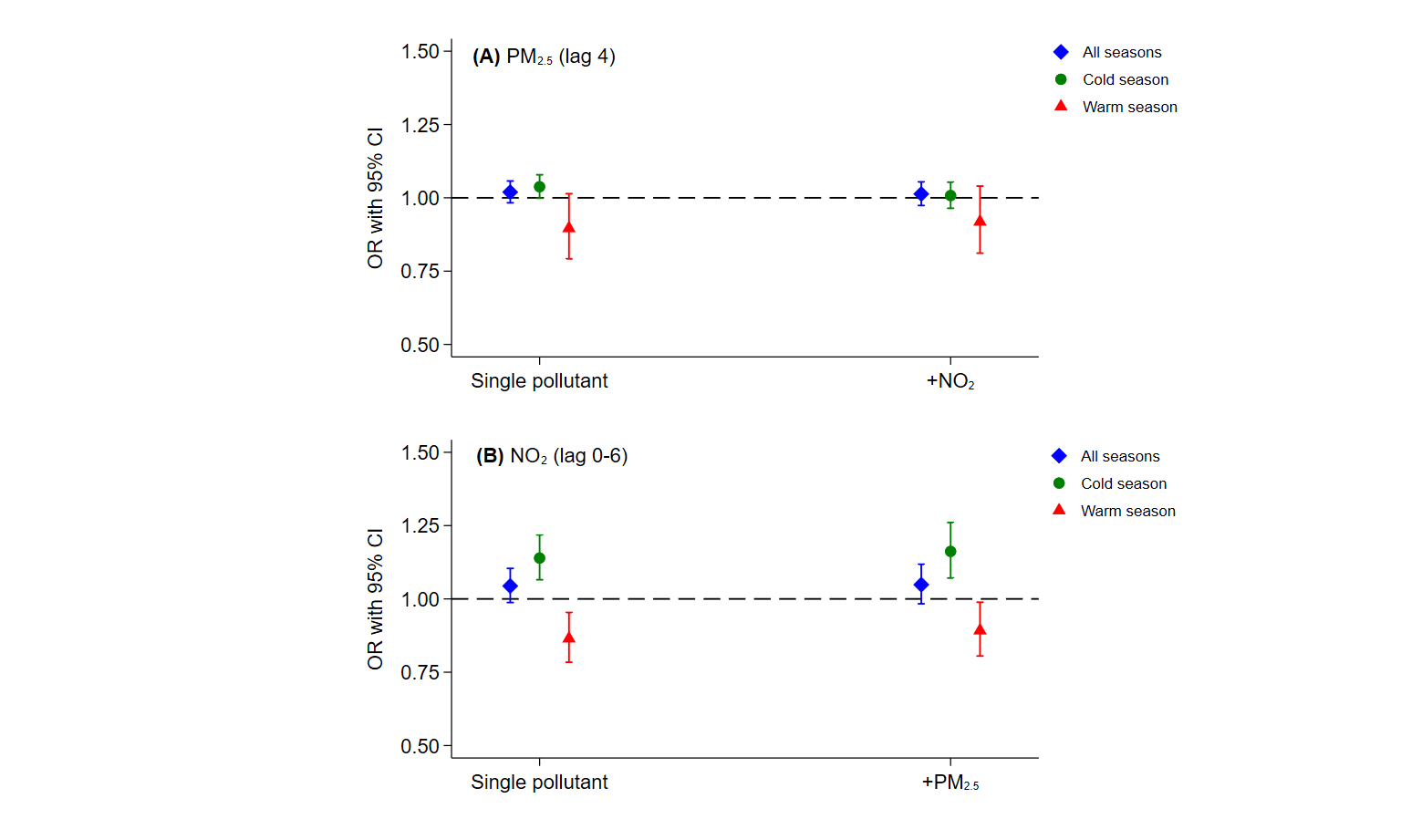


**Fig. S3.** Associations between air pollution and suicide with and without adjustment for co-pollutants. Effect estimates are presented as odds ratios (95%CIs) associated with a 10 µg/m^3^ increase in PM_2.5_ at lag day 6 and a 10 ppb increase in NO_2_ at lag 0-6. Abbreviations: PM_2.5_, particulate matter with aerodynamic diameter ≤2.5 µm; NO_2_, nitrogen dioxide; OR, odds ratio; CI, confidence interval.

**Table S7** Associations of WBGT_max_ and air pollutants with suicide stratified by sex (lag 0-6).

|  | Season | Male |  | Female |
| --- | --- | --- | --- | --- |
| Independent association [OR (95% CI)] |  |  |  |  |
| PM_2.5_ (10 µg/m^3^) ^a^ | All seasons | 1.02 (0.96, 1.08) |  | 0.98 (0.88, 1.09) |
|  | Cold season | 1.04 (0.98, 1.10) |  | 1.03 (0.92, 1.15) |
|  | Warm season | 0.85 (0.68, 1.05) |  | **0.54 (0.36, 0.82)** |
| NO_2_ (10 ppb) ^a^ | All seasons | **1.06 (1.00, 1.13)** |  | 0.98 (0.87, 1.10) |
|  | Cold season | **1.15 (1.07, 1.24)** |  | 1.08 (0.93, 1.25) |
|  | Warm season | **0.88 (0.79, 0.99)** |  | 0.82 (0.67, 1.00) |
| WBGT_max_ (5°C) ^b^ | All seasons | 1.04 (0.99, 1.11) |  | 1.01 (0.91, 1.13) |
|  | Cold season | 0.96 (0.90, 1.04) |  | 0.92 (0.80, 1.05) |
|  | Warm season | **1.22 (1.11, 1.35)** |  | **1.29 (1.06, 1.56)** |
| Multiplicative interaction [OR (95% CI)] |  |  |  |  |
| PM_2.5_ × WBGT_max_ ^c^ | All seasons | 1.00 (0.95, 1.05) |  | **0.90 (0.82, 0.99)** |
|  | Cold season | 1.03 (0.96, 1.11) |  | 1.00 (0.87, 1.16) |
|  | Warm season | 1.16 (0.97, 1.39) |  | 1.02 (0.71, 1.46) |
| NO_2_ × WBGT_max_^d^ | All seasons | 1.02 (0.99, 1.05) |  | 1.02 (0.96, 1.08) |
|  | Cold season | **1.07 (1.02, 1.12)** |  | 1.07 (0.97, 1.17) |
|  | Warm season | **1.15 (1.07, 1.23)** |  | **1.20 (1.05, 1.37)** |
| Additive interaction [RERI (95% CI)] |  |  |  |  |
| PM_2.5_ and WBGT_max_ ^e^ | All seasons | –0.13 (–0.33, 0.07) |  | **–0.47 (–0.81, –0.13)** |
|  | Cold season | –0.23 (–0.62, 0.15) |  | –0.48 (–1.14, 0.18) |
|  | Warm season | **0.33 (0.001, 0.66)** |  | 0.20 (–0.28, 0.68) |
| NO_2_ and WBGT_max_^f^ | All seasons | 0.11 (–0.12, 0.34) |  | –0.16 (–0.53, 0.22) |
|  | Cold season | 0.07 (–0.31, 0.44) |  | –0.50 (–1.14, 0.15) |
|  | Warm season | **0.35 (0.03, 0.67)** |  | **0.45 (0.06, 0.84)** |

Abbreviations: WBGT_max_, maximum wet bulb globe temperature; PM_2.5_, particulate matter with aerodynamic diameter ≤2.5 µm; NO_2_, nitrogen dioxide; OR, odds ratio; CI, confidence interval; RERI, relative excess risk due to interaction.

^a^ This model was adjusted for WBGT_max_.

^b^ This model was adjusted for PM_2.5_.

^c^ Interaction term (WBGT_max_ × PM_2.5_) was added into the two-exposure model to test the interaction between each 5 °C increase in WBGT_max_ and each 10 µg/m^3^ increase in PM_2.5_ on suicide.

^d^ Interaction term (WBGT_max_ × NO_2_) was added into the two-exposure model to test the interaction between each 5 °C increase in WBGT_max_ and each 10 ppb increase in NO_2_ on suicide.

^e^ The 50^th^ percentile of WBGT_max_ (15 °C) and 75^th^ percentile of PM_2.5_ (9 µg/m^3^) were used to classify WBGT_max_ and PM_2.5_ into a binary variable.

^f^ The 50^th^ percentile of WBGT_max_ (15 °C) and 75^th^ percentile of NO_2_ (39 ppb) were used to classify WBGT_max_ and NO_2_ into a binary variable.

A significant interaction term indicates the existence of multiplicative interaction.

A significant RERI indicates the presence of additive interaction.

Statistically significant (*P* <0.05) associations are marked in bold.

**Table S8** Associations of WBGT_max_ and air pollutants with suicide stratified by age (lag 0-6).

|  | Season |  | Age | | |
| --- | --- | --- | --- | --- | --- |
|  |  |  | ≤35 years | 36–64 years | ≥65 years |
| Independent association [OR (95% CI)] |  |  |  |  |  |
| PM_2.5_ (10 µg/m^3^)^a^ | All seasons |  | 1.01 (0.94, 1.09) | 1.02 (0.95, 1.11) | 0.99 (0.85, 1.15) |
|  | Cold season |  | 1.02 (0.95, 1.11) | 1.06 (0.98, 1.15) | 1.02 (0.88, 1.19) |
|  | Warm season |  | 0.88 (0.66, 1.18) | **0.72 (0.55, 0.95)** | **0.45 (0.21, 0.95)** |
| NO_2_ (10 ppb)^a^ |  |  |  |  |  |
|  | All seasons |  | 1.05 (0.97, 1.14) | 1.05 (0.97, 1.14) | 0.98 (0.81, 1.19) |
|  | Cold season |  | **1.12 (1.01, 1.23)** | **1.17 (1.06, 1.30)** | 1.08 (0.86, 1.35) |
|  | Warm season |  | 0.90 (0.77, 1.05) | **0.85 (0.74, 0.97)** | 0.79 (0.56, 1.11) |
| WBGT_max_ (5 °C)^b^ | All seasons |  | 0.99 (0.92, 1.07) | 1.02 (0.95, 1.11) | 1.13 (0.96, 1.33) |
|  | Cold season |  | 0.90 (0.82, 1.00) | 0.96 (0.88, 1.06) | 1.14 (0.93, 1.40) |
|  | Warm season |  | **1.19 (1.04, 1.37)** | **1.29 (1.13, 1.46)** | 1.21 (0.91, 1.61) |
| Multiplicative interaction [OR (95% CI)] |  |  |  |  |  |
| PM_2.5_ × WBGT_max_^c^ | All seasons |  | 1.01 (0.94, 1.08) | 0.97 (0.91, 1.03) | **0.86 (0.74, 0.99)** |
|  | Cold season |  | 1.06 (0.96, 1.17) | 1.03 (0.94, 1.13) | 0.95 (0.77, 1.16) |
|  | Warm season |  | 1.11 (0.87, 1.40) | 1.24 (0.98, 1.57) | 0.68 (0.36, 1.31) |
| NO_2_ × WBGT_max_^d^ | All seasons |  | **1.04 (1.00, 1.09)** | 1.00 (0.96, 1.04) | 0.97 (0.89, 1.06) |
|  | Cold season |  | **1.11 (1.04, 1.19)** | 1.05 (0.99, 1.11) | 0.96 (0.84, 1.10) |
|  | Warm season |  | **1.18 (1.07, 1.30)** | **1.15 (1.05, 1.25)** | 1.15 (0.93, 1.41) |
| Additive interaction [RERI (95% CI)] |  |  |  |  |  |
| PM_2.5_ and WBGT_max_^e^ | All seasons |  | –0.19 (–0.45, 0.07) | –0.18 (–0.42, 0.06) | –0.50 (–1.14, 0.15) |
|  | Cold season |  | –0.39 (–0.86, 0.08) | –0.19 (–0.69, 0.30) | 0.34 (–1.46, 2.13) |
|  | Warm season |  | 0.38 (–0.04, 0.81) | 0.34 (–0.01, 0.68) | –1.58 (–5.04, 1.90) |
| NO_2_ and WBGT_max_^f^ | All seasons |  | 0.08 (–0.20, 0.36) | 0.05 (–0.24, 0.35) | –0.29 (–1.02, 0.44) |
|  | Cold season |  | 0.16 (–0.32, 0.65) | –0.12 (–0.60, 0.36) | –0.79 (–1.97, 0.39) |
|  | Warm season |  | **0.41 (0.08, 0.75)** | 0.35 (–0.05, 0.76) | 0.29 (–0.66, 1.25) |

Abbreviations: WBGT_max_, maximum wet bulb globe temperature; PM_2.5_, particulate matter with aerodynamic diameter ≤2.5 µm; NO_2_, nitrogen dioxide; OR, odds ratio; CI, confidence interval; RERI, relative excess risk due to interaction

^a^ This model was adjusted for WBGT_max_.

^b^ This model was adjusted for PM_2.5_.

^c^ Interaction term (WBGT_max_ × PM_2.5_) was added into the two-exposure model to test the interaction between each 5 °C increase in WBGT_max_ and each 10 µg/m^3^ increase in PM_2.5_ on suicide.

^d^ Interaction term (WBGT_max_ × NO_2_) was added into the two-exposure model to test the interaction between each 5 °C increase in WBGT_max_ and each 10 ppb increase in NO_2_ on suicide.

^e^ The 50^th^ percentile of WBGT_max_ (15 °C) and 75^th^ percentile of PM_2.5_ (9 µg/m3) were used to classify WBGT_max_ and PM_2.5_ into a binary variable.

^f^ The 50^th^ percentile of WBGT_max_ (15 °C) and 75^th^ percentile of NO_2_ (39 ppb) were used to classify WBGT_max_ and NO_2_ into a binary variable.

A significant interaction term indicates the existence of multiplicative interaction.

A significant RERI indicates the presence of additive interaction.

Statistically significant (*P* <0.05) associations are marked in bold.

**Table S9** Associations of WBGT_max_ and air pollutants with suicide stratified by region (lag 0-6).

|  | Season |  | Region | | |
| --- | --- | --- | --- | --- | --- |
|  |  |  | SL County |  | Non-SL County |
| Independent association [OR (95% CI)] |  |  |  |  |  |
| PM_2.5_ (10 µg/m^3^) ^a^ | All seasons |  | 1.04 (0.97, 1.12) |  | 0.98 (0.91, 1.05) |
|  | Cold season |  | 1.06 (0.99, 1.14) |  | 1.01 (0.93, 1.08) |
|  | Warm season |  | 0.78 (0.58, 1.05) |  | **0.75 (0.59, 0.97)** |
| NO_2_ (10 ppb)^a^ | All seasons |  | **1.11 (1.02, 1.21)** |  | 0.99 (0.92, 1.07) |
|  | Cold season |  | **1.22 (1.10, 1.35)** |  | 1.08 (0.98, 1.18) |
|  | Warm season |  | 0.93 (0.81, 1.08) |  | **0.82 (0.72, 0.94)** |
| WBGT_max_ (5 °C)^b^ | All seasons |  | 1.07 (0.99, 1.16) |  | 1.01 (0.95, 1.08) |
|  | Cold season |  | 0.99 (0.89, 1.09) |  | 0.93 (0.86, 1.01) |
|  | Warm season |  | **1.27 (1.11, 1.46)** |  | **1.21 (1.08, 1.36)** |
| Multiplicative interaction [OR (95% CI)] |  |  |  |  |  |
| PM_2.5_ × WBGT_max_ ^c^ | All seasons |  | 0.97 (0.90, 1.03) |  | 0.98 (0.92, 1.04) |
|  | Cold season |  | 1.00 (0.91, 1.10) |  | 1.04 (0.95, 1.14) |
|  | Warm season |  | 1.18 (0.92, 1.50) |  | 1.09 (0.88, 1.36) |
| NO_2_ × WBGT_max_^d^ | All seasons |  | 1.01 (0.97, 1.06) |  | 1.01 (0.98, 1.05) |
|  | Cold season |  | 1.06 (0.98, 1.14) |  | **1.07 (1.01, 1.12)** |
|  | Warm season |  | **1.25 (1.13, 1.39)** |  | **1.11 (1.03, 1.20)** |
| Additive interaction [RERI (95% CI)] |  |  |  |  |  |
| PM_2.5_ and WBGT_max_ ^e^ | All seasons |  | **–0.34 (–0.60, –0.07)** |  | –0.12 (–0.35, 0.10) |
|  | Cold season |  | –0.29 (–0.79, 0.20) |  | –0.31 (–0.77, 0.14) |
|  | Warm season |  | 0.12 (–0.31, 0.55) |  | **0.45 (0.11, 0.78)** |
| NO_2_ and WBGT_max_^f^ | All seasons |  | –0.05 (–0.34, 0.24) |  | 0.13 (–0.15, 0.40) |
|  | Cold season |  | –0.08 (–0.56, 0.40) |  | –0.03 (–0.48, 0.41) |
|  | Warm season |  | 0.33 (–0.04, 0.69) |  | **0.43 (0.07, 0.79)** |

Abbreviations: SL, Salt Lake; WBGT_max_, maximum wet bulb globe temperature; PM_2.5_, particulate matter with aerodynamic diameter ≤2.5 µm; NO_2_, nitrogen dioxide; OR, odds ratio; CI, confidence interval; RERI, relative excess risk due to interaction.

^a^ This model was adjusted for WBGT_max_.

^b^ This model was adjusted for PM_2.5_.

^c^ Interaction term (WBGT_max_ × PM_2.5_) was added into the two-exposure model to test the interaction between each 5 °C increase in WBGT_max_ and each 10 µg/m^3^ increase in PM_2.5_ on suicide.

^d^ Interaction term (WBGT_max_ × NO_2_) was added into the two-exposure model to test the interaction between each 5 °C increase in WBGT_max_ and each 10 ppb increase in NO_2_ on suicide.

^e^ The 50^th^ percentile of WBGT_max_ (15 °C) and 75^th^ percentile of PM_2.5_ (9 µg/m3) were used to classify WBGT_max_ and PM_2.5_ into a binary variable.

^f^ The 50^th^ percentile of WBGT_max_ (15 °C) and 75^th^ percentile of NO_2_ (39 ppb) were used to classify WBGT_max_ and NO_2_ into a binary variable.

A significant interaction term indicates the existence of multiplicative interaction.

A significant RERI indicates the presence of additive interaction.

Statistically significant (*P* <0.05) associations are marked in bold.

**Table S10** Odds ratios of suicide mortality associated with each 5 °C increase in WBGT_max_ and 10 unit increase in PM_2.5_ and NO_2_ from single- and two-exposure models (lag 0-6).

| Models | Exposure | Winter |  | Summer |
| --- | --- | --- | --- | --- |
|  |  | OR (95% CI) |  | OR (95% CI) |
| Single-exposure model^a^ | PM_2.5_ | 1.04 (0.98, 1.10) |  | 1.07 (0.87, 1.32) |
|  | NO_2_ | **1.17 (1.07, 1.28)** |  | 0.98 (0.85, 1.13) |
|  | WBGT_max_ | **0.81 (0.73, 0.89)** |  | **1.90 (1.63, 2.21)** |
|  |  |  |  |  |
| Two-exposure model  (PM_2.5_ and WBGT_max_)^b^ | PM_2.5_ | **1.06 (1.00, 1.12)** |  | 0.87 (0.69, 1.09) |
|  | WBGT_max_ | **0.79 (0.72, 0.88)** |  | **1.94 (1.66, 2.27)** |
|  |  |  |  |  |
| Two-exposure model  (NO_2_ and WBGT_max_)^c^ | NO_2_ | **1.19 (1.09, 1.30)** |  | 0.93 (0.80, 1.07) |
|  | WBGT_max_ | **0.80 (0.72, 0.88)** |  | **1.91 (1.64, 2.23)** |

Abbreviations: PM_2.5_, particulate matter with aerodynamic diameter ≤2.5µm; NO_2_, nitrogen dioxide; WBGT_max_, maximum wet bulb globe temperature; OR, odds ratio; CI, confidence interval.

^a^ This model included only WBGT_max,_ PM_2.5,_ or NO_2_ to test the main effect of each 5 °C increase in WBGT_max_ or 10 unit increase in PM_2.5_ or NO_2_ on suicide mortality.

^b^ This model included WBGT_max_ and PM_2.5_ simultaneously to test the main effects of each 5 °C increase in WBGT_max_ and each 10 µg/m^3^ increase in PM_2.5_ on suicide mortality.

^c^ This model included WBGT_max_ and NO_2_ simultaneously to test the main effects of each 5 °C increase in WBGT_max_ and each 10 ppb increase in NO_2_ on suicide mortality.

Statistically significant (*P* <0.05) associations are marked in bold.

**Table S11** Odds ratio and 95% confidence intervals for suicide mortality associated with a 5 ℃ increase in WBGT_max_ at different air pollution levels (lag 0-6).

| Modifier | Air pollution levels^a^ | All seasons |  | Cold season |  | Warm season |
| --- | --- | --- | --- | --- | --- | --- |
|  |  | OR (95% CI |  | OR (95% CI) |  | OR (95% CI) |
| PM_2.5_ | Low (< P25) | 1.02 (0.90, 1.15) |  | 0.94 (0.81, 1.10) |  | 1.15 (0.94, 1.40) |
|  | Moderate (P25–75) | 1.08 (0.98, 1.18) |  | 0.98 (0.86, 1.11) |  | **1.19 (1.05, 1.35)** |
|  | High (> P75) | 0.95 (0.84, 1.06) |  | 0.94 (0.83, 1.06) |  | 1.03 (0.63, 1.69) |
|  |  |  |  |  |  |  |
| NO_2_ | Low (< P25) | 0.97 (0.86, 1.09) |  | 0.87 (0.74, 1.03) |  | 1.07 (0.90, 1.27) |
|  | Moderate (P25–75) | **1.13 (1.05, 1.22)** |  | 1.03 (0.93, 1.14) |  | **1.29 (1.15, 1.45)** |
|  | High (> P75) | 0.96 (0.86, 1.06) |  | 0.89 (0.80, 1.00) |  | **1.43 (1.08, 1.59)** |

Abbreviations: PM_2.5_, particulate matter with aerodynamic diameter ≤2.5 µm; NO_2_, nitrogen dioxide; WBGT_max_, maximum wet bulb globe temperature; OR, odds ratio; CI, confidence interval.

^a^ Definition of air pollution level: low, moderate, and high levels defined as below 25^th^ (P25), between 25th and 75^th^ (P25–75), and above 75^th^ (P75) percentiles of PM_2.5_ and NO_2_ levels, respectively.

Lag 0-6 was considered for both air pollution and WBGT_max_.

Statistically significant (*P* <0.05) associations are marked in bold.

**Table S12** Odds ratios of suicide mortality associated with each 10 unit increase in PM_2.5_ and NO_2_ on moving-average lag days during the warm season, excluding data from Independence Day (July 4th).

| Models | Exposure | Lag 0-1 | Lag 0-3 | Lag 0-6 |
| --- | --- | --- | --- | --- |
|  |  | OR (95% CI) | OR (95% CI) | OR (95% CI) |
| Unadjusted model^a^ | PM_2.5_ | **0.83 (0.73, 0.95)** | **0.84 (0.72, 0.98)** | 0.86 (0.71, 1.03) |
|  | NO_2_ | 0.95 (0.89, 1.01) | 0.93 (0.86, 1.01) | **0.89 (0.81, 0.98)** |
|  |  |  |  |  |
| Adjusted model^b^ | PM_2.5_ | **0.76 (0.66, 0.88)** | **0.76 (0.64, 0.90)** | **0.76 (0.63, 0.92)** |
|  | NO_2_ | **0.92 (0.86, 0.98)** | **0.90 (0.83, 0.98)** | **0.86 (0.78, 0.95)** |

Abbreviations: PM_2.5_, particulate matter with aerodynamic diameter ≤2.5 µm; NO_2_, nitrogen dioxide; OR, odds ratio; CI, confidence interval.

^a^ This model included only PM_2.5_ or NO_2_.

^b^ This model was adjusted for WBGT_max_.

Statistically significant (*P* <0.05) associations are marked in bold.

**Table S13** E-values (95% Confidence Interval) at odds ratio scale for the estimation of associations in main estimates (lag 0-6).

| Exposure | Season | OR (95% CI)^a^ |  | E-value (rare outcome)  OR (LCL) |
| --- | --- | --- | --- | --- |
| PM_2.5_ (10 µg/m^3^) | All seasons | 1.01 (0.96, 1.07) |  | 1.10 (1.00) |
|  | Cold season | 1.04 (0.99, 1.09) |  | 1.24 (1.00) |
|  | Warm season | 0.76 (0.63, 0.92) |  | 1.96 (1.39) |
|  |  |  |  |  |
| NO_2_ (10 ppb) | All seasons | 1.04 (0.99, 1.10) |  | 1.24 (1.00) |
|  | Cold season | 1.14 (1.07, 1.22) |  | 1.54 (1.34) |
|  | Warm season | 0.86 (0.78, 0.95) |  | 1.60 (1.29) |
|  |  |  |  |  |
| WBGT_max_ (5 °C) | All seasons | 1.03 (0.98, 1.09) |  | 1.21 (1.00) |
|  | Cold season | 0.95 (0.89, 1.01) |  | 1.29 (1.00) |
|  | Warm season | 1.22 (1.12, 1.33) |  | 1.74 (1.49) |

Abbreviations: PM_2.5_, particulate matter with aerodynamic diameter ≤2.5 µm; NO_2_, nitrogen dioxide; WBGT_max_, maximum wet bulb globe temperature; OR, odds ratio; CI, confidence interval; LCL, lower 95% confidence limit.

E-value is defined as the minimum strength of association on the odds ratio scale that an unmeasured confounder would need to have with the exposure and the outcome to fully explain away a specific exposure-outcome association, conditional on the measured covariates. (VanderWeele, TJ & Ding P. Sensitivity analysis in observational research: introducing the E-value. Annals of internal medicine 2017;167(4):268-274). Calculated with reference to online calculator: <https://www.evalue-calculator.com/evalue/>. Rare outcome refers to outcome occurring <15% in the main population.

^a^ Obtained from the two-exposure models included in the main text (Table 3).

**References**

1. Ahn Y, Tuholske C, Parks RM. Comparing approximated heat stress measures across the United States. Geohealth. 2024;8(1):e2023GH000923.
2. Liljegren JC, Carhart RA, Lawday P, Tschopp S, Sharp R. Modeling the wet bulb globe temperature using standard meteorological measurements. J Occup Environ Hyg. 2008;5(10):645–555.
